# Persistence and Change in Community Composition of Reef Corals through Present, Past, and Future Climates

**DOI:** 10.1371/journal.pone.0107525

**Published:** 2014-10-01

**Authors:** Peter J. Edmunds, Mehdi Adjeroud, Marissa L. Baskett, Iliana B. Baums, Ann F. Budd, Robert C. Carpenter, Nicholas S. Fabina, Tung-Yung Fan, Erik C. Franklin, Kevin Gross, Xueying Han, Lianne Jacobson, James S. Klaus, Tim R. McClanahan, Jennifer K. O'Leary, Madeleine J. H. van Oppen, Xavier Pochon, Hollie M. Putnam, Tyler B. Smith, Michael Stat, Hugh Sweatman, Robert van Woesik, Ruth D. Gates

**Affiliations:** 1 Department of Biology, California State University Northridge, Northridge, California, United States of America; 2 Institut de Recherche pour le Développement, Unité de Recherche CoReUs, Observatoire Océanologique de Banyuls, Banyuls-sur-Mer, France; 3 Laboratoire d'Excellence "CORAIL", Perpignan, France; 4 Department of Environmental Science and Policy, University of California Davis, Davis, California, United States of America; 5 Department of Biology, The Pennsylvania State University, University Park, Pennsylvania, United States of America; 6 Department of Earth and Environmental Sciences, University of Iowa, Iowa City, Iowa, United States of America; 7 Center for Population Biology, University of California Davis, Davis, California, United States of America; 8 National Museum of Marine Biology and Aquarium, Taiwan, Republic of China; 9 Hawaii Institute of Marine Biology, School of Ocean and Earth Science and Technology, University of Hawaii, Kaneohe, Hawaii, United States of America; 10 Biomathematics Program, North Carolina State University, Raleigh, North Carolina, United States of America; 11 Department of Ecology, Evolution and Marine Biology and the Coastal Research Center, Marine Science Institute, University of California Santa Barbara, Santa Barbara, California, United States of America; 12 National Center for Ecological Analysis and Synthesis, Santa Barbara, California, United States of America; 13 Department of Biology, University of Florida, Gainesville, Florida, United States of America; 14 Department of Geological Sciences, University of Miami, Coral Gables, Florida, United States of America; 15 Wildlife Conservation Society, Marine Program, Bronx, New York, United States of America; 16 Australian Institute of Marine Science, Townsville, Queensland, Australia; 17 The Cawthron Institute, Nelson, New Zealand; 18 Center for Marine and Environmental Studies, University of the Virgin Islands, St. Thomas, Virgin Islands, United States of America; 19 The University of Western Australia Oceans Institute and the Centre for Microscopy, Characterisation and Analysis, University of Western Australia, Crawley, Western Australia, Australia; 20 Department of Biological Sciences, Florida Institute of Technology, Melbourne, Florida, United States of America; College of Charleston, United States of America

## Abstract

The reduction in coral cover on many contemporary tropical reefs suggests a different set of coral community assemblages will dominate future reefs. To evaluate the capacity of reef corals to persist over various time scales, we examined coral community dynamics in contemporary, fossil, and simulated future coral reef ecosystems. Based on studies between 1987 and 2012 at two locations in the Caribbean, and between 1981 and 2013 at five locations in the Indo-Pacific, we show that many coral genera declined in abundance, some showed no change in abundance, and a few coral genera increased in abundance. Whether the abundance of a genus declined, increased, or was conserved, was independent of coral family. An analysis of fossil-reef communities in the Caribbean revealed changes in numerical dominance and relative abundances of coral genera, and demonstrated that neither dominance nor taxon was associated with persistence. As coral family was a poor predictor of performance on contemporary reefs, a trait-based, dynamic, multi-patch model was developed to explore the phenotypic basis of ecological performance in a warmer future. Sensitivity analyses revealed that upon exposure to thermal stress, thermal tolerance, growth rate, and longevity were the most important predictors of coral persistence. Together, our results underscore the high variation in the rates and direction of change in coral abundances on contemporary and fossil reefs. Given this variation, it remains possible that coral reefs will be populated by a subset of the present coral fauna in a future that is warmer than the recent past.

## Introduction

Most present-day coral reefs differ from the reefs that were first described by ecologists and explorers [1], and recent evidence suggests that the rate of change in environmental factors affecting coral survival is accelerating as a result of global climate change (GCC) and ocean acidification (OA) [2]. Many coral reefs have changed dramatically in benthic community structure over the last few decades [3], but contemporary research has focused on declining abundances of scleractinian corals rather than on the few cases where reefs have retained coral cover (or recovered following losses), and where some scleractinian corals have maintained or increased in abundance [4]–[6].

Coral reefs in remote settings provide some of the best examples of reefs with high coral cover and intact trophic structures [6]–[8], and their distance from localized anthropogenic effects suggest isolation and protection, rather than global climate, are determinants of their present condition. In addition, coral reefs with diverse scleractinian faunas and relatively high coral cover also can be found in marginal locations characterized by high temperature fluctuations [9], thermal extremes [10], and turbidity [11]. Moreover, while many coral genera have declined in abundance, some persist in ecologically dominant roles, which have led to the suggestion that corals on contemporary reefs can be categorized as “losers” or “winners” [12]. Massive *Porites* spp. is an example of a group of corals that is faring better than others and is increasing in abundance in the Pacific and Caribbean [4], [13], and also is showing signs of resistance to OA, both in mesocosms [14] and in at least one reef environment where volcanic carbon dioxide seeps into the seawater [15].

Considerable effort is being dedicated to elucidating the processes driving shifts in coral community structure on contemporary reefs, and characterizing the biological and ecological traits of scleractinian corals that are resistant to disturbances [16], [17]. Information is still needed to advance this effort, for example, to evaluate whether shifts in coral community structure are a result of reduced coral recruitment, increased mortality of adult corals, or both. These and other processes interact to determine the trajectories of change in the composition of coral communities. For instance, with increased coral mortality driving regional reductions in fecundity and population size, coral recruitment likely will decline and create compensatory density dependence favoring further reductions in coral cover. Such population-level events are also affected by processes such as herbivory, predation, regional oceanography, and climate change, which alter coral reef communities over short periods. Over geologic time, macroprocesses such as ice ages cause changes in the composition of coral reef communities that are captured in fossilized reefs, where the success of coral species may be discovered based on their retention (or loss) from the fossil record [18]. The fossil record therefore provides a tool through which it is possible to analyze how corals responded to environmental or biological changes in the past, and over much longer time frames than is covered by ecological studies.

The goals of this study were to use long-term data from modern and fossil coral reefs to test for variation among coral genera in the rates and directions of change in abundance over time, to use these trends to consider which genera have the potential to persist as seawater warms through climate change, and to evaluate in what form these genera might assemble in the future to form coral communities. To achieve these goals, we synthesized data from extant reefs at seven locations (“case studies”) and from fossil coral reef communities in the Caribbean, and developed a mathematical model to evaluate which traits are most likely advantageous in promoting persistence of coral genera in warming oceans. We present our analyses in three parts: first, we describe the events taking place on extant reefs by examining aspects of ecological records from our case studies (i.e., the Present); second, we use the fossil record (i.e., the Past) to gain insight into the temporal novelty of the changes affecting the community ecology of extant reefs, and whether clues to the ultimate outcomes of these changes might be found in the past; finally, we use a mathematical model to offer insight into the potential ecological fate of coral reefs under increased thermal stress (i.e., the Future).

## Present

Recent efforts to describe changes in the composition of coral reef communities have typically focused on scleractinian corals and their performance relative to other functional groups such as macroalgae [19]. These efforts have brought attention to the large losses of coral cover that have taken place since the 1960s [20]. It is uncommon however, for such studies to explicitly focus on the extent to which coral taxa differ in the way they respond to climate change, a characteristic that could play an important role in determining the future community structure of coral reefs [21]. One example of the value of such approaches is the analysis of coral bleaching on the reefs of Okinawa in 1998, the results of which allowed corals to be categorized based on whether they survived (i.e., winners) or died (i.e., losers) on the short-term following the disturbance [12]. Analysis of the same community over 14 years revealed discrepancies between short-term and long-term winners, both in the trajectories of changing abundance as well as in the demographic mechanisms underpinning those trajectories [22]. Nonetheless, understanding of the community dynamics of a reef in Okinawa was well served by considering variability in the response of corals to a disturbance. In the first portion of our analysis, we focused on long-term trajectories of change in cover of scleractinian corals at several well-studied locations that represent case-studies for the present study, and sought to determine the extent to which these trajectories differed among coral genera. Ecological data for coral genera at two Caribbean and five Indo-Pacific locations were used to explore changes in absolute and relative coral cover over time.

To support our analysis, data were gathered from nine projects in seven locations where multiple sites have been censused frequently, and together span up to 33 y (1981–2013, although not all studies were of equal length) (Table S1 in File S1). The present authors either collected these data (for the US Virgin Islands, Belize, Kenya, Moorea [2005–2010], and Taiwan), or were directly associated with the agencies that collected the data. Most data came from shallow reefs (≤10 m depth), with some from 17 m depth (Moorea), 11–25 m depth (parts of the US Virgin Islands), or>25 m depth (northern US Virgin Islands) (Table S1 in File S1). For all locations, except the Great Barrier Reef (GBR, Australia), data were averaged across sites on a scale of ∼10 km. Data from the GBR posed special challenges because it encompassed a large number of sites representing an extensive area (>150,000 km^2^) that would individually have considerable leverage on the analysis. The GBR data were therefore collapsed into three habitats - inshore (11 reefs), mid shelf (18 reefs), and outer shelf (18 reefs) - and pooled among latitudes. For all sites, data were summarized annually as percentage cover of scleractinian corals by genus, using taxonomy as described in recent papers [23], [24].

The rate of change in coral cover by genus over the duration of each study was evaluated using least-squares linear regression, with analyses separated for the Caribbean (two locations) and Indo-Pacific (five locations) (Table S2 in File S1). Changes in abundance by genus were expressed on absolute and relative scales, with relative cover determined by dividing the cover for each genus by the total coral cover at the study location at the same time. Regression slopes for each coral taxon (i.e., change in coral cover over time, % y^−1^) were used in subsequent analyses, and slopes were used regardless of their statistical significance. While the significance of any one slope can be evaluated with *P* values based on the ratio of the mean sum of squares explained by the regression and unexplained variance, in data compilations such as used in the present study, the least squares estimate of the slope is an unbiased estimate of the true slope that is preferable to the biased slope estimate derived by assuming non-significant slopes have a value of zero. The frequency distribution of these slopes would then be distributed more uniformally than one in which non-significant slopes were set to zero, which would create an ecologically unrealistic gap between actual zero slopes and the larger slopes that are statistically significant. Having calculated the slopes of the relationships between coral cover and time, the frequency distributions of the slopes were tested for skewness using a g_2_ test, and for normality using a Kolmogorov-Smirnov (K-S) test. Our objective was to understand how corals were responding to the combined effects of biotic and abiotic disturbances extending over multiple decades, and we did not intend to partition these changes to the effects of individual pulse or press disturbances. Such disturbances might reflect rapidly-acting events such as severe storms, or a large-scale predator outbreaks (for example, the corallivorous seastar *Acanthaster planci*), or chronic effects such as rising seawater temperature or declining seawater pH. Therefore the rates of change in coral cover we report cannot be attributed to specific causal processes and cannot be used to distinguish between the response of corals to recent dramatic and local events versus long-lasting, chronic, and regional-scale events. Instead, our analyses attempted to ‘capture’ the culmination of the above-mentioned processes and events as time-averaged rates of change in cover of each coral genus.

The changes in cover over time in genus-level coral abundance on absolute and relative scales were used to test the hypotheses that: (1) changes in abundance were independent of overall dominance in the community, meaning that abundant and rare coral genera were likely to share similar trajectories of change; and (2) the covariance between changes in absolute and relative abundance was random with regard to distributing coral genera on these axes, meaning that coral genera were equally likely to have any fate defined by all possible combinations of changes in absolute and relative abundances. These analyses were conducted to evaluate the effect of abundance on change in relative abundance (i.e., a measure of success), and were designed so that they could be completed for fossil data as well as ecological data from our case-study locations. Dominance was evaluated as the rank abundance by genus across the entire data set, and the relationship between abundance and success was evaluated separately for our case-history sites from the Caribbean and Indo-Pacific.

The associations between change in relative abundance and rank dominance were tested using Pearson correlations, first by genus, and second by family. The two analyses were used to evaluate the extent to which the dominance–success relationships were independent of taxon. The analyses of covariance between absolute and relative abundance were used to identify genera that had increased in absolute and relative cover, and to evaluate regional variation in these characteristics. Based on the ratio of the change in absolute to relative coral cover, coral genera were separated into four domains with differing trajectories of change in cover: (1) S-domain corals showed the *s*trongest ecological performance by increasing cover on both absolute and relative scales; (2) M-domain corals showed *m*oderate ecological performance by increasing in absolute cover but declining in relative cover, because other taxa increased faster still; (3) W-domain corals showed *w*eak ecological performance by declining in absolute cover but increasing in relative cover, because other taxa declined in coral cover at a faster rate; and (4) F-domain corals showing *f*ailing ecological performance by decreasing in cover on both absolute and relative scales. The covariance between changes in absolute and relative abundance was analyzed with Pearson correlations to test for a random association between variables. Rejection of the null hypothesis would indicate a positive or negative association. Additionally, the distribution of the coral genera among the S-, M-, W- and F- domains was tested for equality using a χ^2^ test.

**Figure 1:**
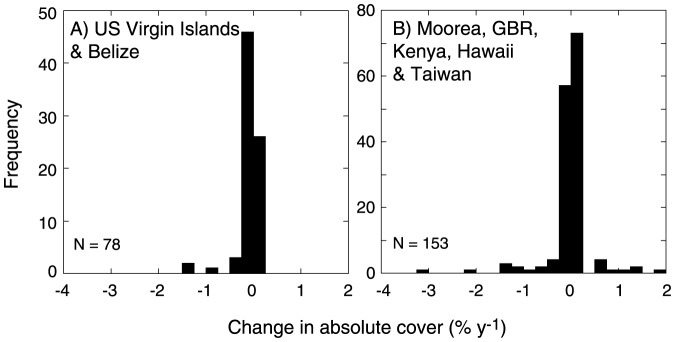
Frequency distributions of changes in absolute coral cover by genus between 1987 and 2012 (two Caribbean sites) and 1987 and 2013 (five Indo-Pacific sites). (A) US Virgin Islands and Belize, and (B) Moorea, Hawaii, Taiwan, the Great Barrier Reef, and Kenya; sample sizes (*n*) shown in all panels. See http://knb.ecoinformatics.org/knb/metacat/nceas.973/knb for raw data, and Table S3 for changes in coral abundances described by these distributions.

Our compilation generated 78 trajectories of changing coral cover by genus from the US Virgin Islands and Belize in the Caribbean, and 153 trajectories from Moorea, Hawaii, Taiwan, Kenya, and the GBR in the Indo-Pacific (Table S2 in File S1). The frequency distributions of changes in absolute coral cover were leptokurtic (based on the statistic g_2_ ≥14.9 [25]), centered on stable cover (i.e., 0% y^−1^), and departed significantly from a normal distribution (K-S *D* statistic ≥0.790, *P*<0.001) (Fig. 1). At least 46% of trajectories in each region showed declines in cover.

Overall, coral cover represented 17 coral families (Table S2 in File S1), and when analyzed by location and habitat, the changes in absolute cover (mean ± SD) ranged from −0.573±0.502% y^−1^ (*Orbicella annularis* complex, n = 9) to 0.004±0.005% y^−1^ (*Stephanocoenia*, n = 6) for the Caribbean, and from −0.439±1.056% y^−1^ (*Montipora*, n = 11) to 0.638±0.004% y^−1^ (*Dipsastrea*, n = 3) for the Indo-Pacific (all mean ± SD). On a relative scale, the range of changes were -0.149±0.319% y^−1^ (*Orbicella*, n = 9) to 0.172±0.236% y^−1^ (*Porites*, n = 9) for the Caribbean, and ranged from −0.869±1.572% y^−1^ (*Acropora*, n = 10) to 1.274±2.695% y^−1^ (*Dipsastrea*, n = 3) for the Indo-Pacific. Mean coral abundance by genus, averaged across all study locations and dates within each region, ranged from 0.002% (*Mussa*) to 15.538% (*Orbicella*) for the Caribbean, and from 0.002% (*Stylocoeniella*) to 7.031% (*Acropora*) for the Indo-Pacific. The regions were characterized by 16 and 41 coral genera respectively, with these representing 9 families in the Caribbean and 12 families in the Indo-Pacific. Project-wide mean coral covers were used to establish a ranking scheme for the abundance of coral genera and families in each location (Table S4 in File S1). For both regions, abundance (i.e., high dominance) of coral genera was associated with more extreme trajectories of relative abundance (Fig. S1 in File S1), although neither relationship was significant (r≤ |0.080|, df≤39, *P*≥0.619). Analysis of these relationships at a high taxonomic level was problematic because of the limited replication of genera within each family, although this was accomplished for Meandrinidae (n = 3 genera) and Mussidae (n = 6 genera) in the Caribbean, and Acroporidae (n = 4 genera), Agariciidae (n = 3 genera), Lobophylliidae (n = 5 genera), Merulinidae (n = 12 genera), Pocilloporidae (n = 5 genera) and Poritidae (n = 4 genera) in the Indo-Pacific. For the Caribbean, the trajectories of change in relative cover did not vary between families (U = 16, *P* = 0.071), and for the Mussidae, they were unrelated to relative dominance in the community (r = −0.556, df = 4, *P*>0.050) (Fig. S1 in File S1). For the Indo-Pacific, the trajectories of change in relative cover also did not vary among families (H = 9.238, *P* = 0.100), and for the Acroporidae, Lobophylliidae, Merulinidae, Pocilloporidae, and Poritidae, were unrelated to relative dominance in the community (r<0.692, 10≥df≥2, *P*>0.050) (Fig. S1 in File S1).

**Figure 2:**
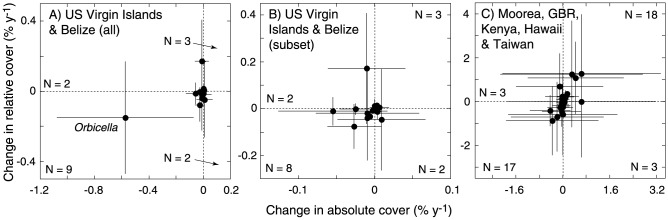
Scatterplots displaying changes in relative abundance (ordinates) and changes in absolute abundance (abscissas) for scleractinian corals in the Caribbean (A and B) and Indo-Pacific (C). Data show mean ± SD (where *n*>1) on both abundance scales for genus [Table S4 in File S1]); N =  sample sizes for each quadrant. (A) Caribbean scaled to show *Orbicella*, (B) Caribbean scaled to show taxa other than *Orbicella*, and (C) Indo-Pacific; axes vary among plots. The two axes of these plots define a two-dimensional performance space separated into four quadrats: S-domain corals (top right, increasing on both absolute and relative scales), M-domain corals (top left, increasing in absolute cover, but declining in relative cover), W-domain corals (bottom right, declining in absolute cover, increasing in relative cover), and F-domain corals (bottom left, declining in both absolute and relative cover).

Analyses of the covariation between relative and absolute cover by genus (Table S3 in File S1) revealed relationships (Fig. 2) that departed significantly from random for the Caribbean (r = 0.575, df = 14, *P*<0.050) and Indo-Pacific (r = 0.707, df = 39, *P*<0.010). Overall, there were positive relationships between the rates of change in relative and absolute cover, showing that there were more genera in the S- and F- domains than chance alone would predict. This outcome was consistent with the distribution of genera among the F-, M-, S- and W- domains, which departed significantly from an expectation of equal representations for the Caribbean (χ^2^ = 8.5, df = 3, *P*≤0.05) and Indo-Pacific (χ ^2^ = 20.56, df = 3, *P*≤0.001); in these cases, significance was a result of the relatively large number of genera in F-domain for the Caribbean and in the F- and S- domains for the Indo-Pacific. S- and F- domain corals were found in the Caribbean and in the Indo-Pacific, but ≤3 genera were categorized as M- or W-domain corals in either region (Table S3 in File S1). The trajectories of changing coral cover in this two-dimensional space (Fig. 2) were clustered around the origin for the two Caribbean locations – showing that most genera changed little over the study period; trajectories were more divergent for the five Indo-Pacific locations. Additionally, trajectories were consistent in the Caribbean (i.e., SDs based on site replicates for genera were small) and more variable in the Indo-Pacific (i.e., SDs were large), although these differences in SDs were not statistically significant (U = 121, n_1_ = 10, n_2v_ = 21, *P* = 0.302).

Our results from reefs in seven locations revealed stasis in genus-level coral cover (i.e., near-zero absolute change) rather than unequivocal declines [2], [3], [26], and a high degree of among-genus variation in response to the combined biotic and abiotic drivers of change that have affected these reefs over the last few decades. As has recently been suggested [21], these outcomes are likely to be important in evaluating the composition of coral communities that might persist in these locations in the future. Our analyses have a number of limitations, notably that region-wide inferences are based on only a small number of locations. Further, the temporal trends were constrained by the time period of sampling at each case-study site, which lead to haphazard sampling of biotic and abiotic disturbances affecting coral cover, and to the exclusion of important events that occurred outside of the sampling period. In the Caribbean, our sampling also occurred after large declines in coral cover had already occurred [26], and after the regional near-extirpation of *Acropora* spp. [27].

In summary, our analyses of coral communities at seven locations describe trends that provide higher resolution details of the large-scale declines of coral cover that have been reported elsewhere [3], [28], notably demonstrating that absolute and relative cover of many coral genera have at least remained relatively unchanged at least over the last few decades. It is important to note, however, that our analyses do not imply a ‘rosy’ future for tropical coral reefs: the future of many coral genera remains uncertain, and relatively few S-domain corals display a strong capacity to increase in cover (Fig. 2). Overall, our genus-level analysis revealed that: (1) many corals have changed little in cover over the last 20–30 y, (2) the absolute and relative cover of a few genera, like *Orbicella* in the US Virgin Islands and Belize and *Pocillopora* and *Acropora* in Moorea, the GBR, Kenya, Hawaii, and Taiwan, have declined rapidly, and (3) the absolute or relative covers of only a few coral genera have increased.

## Past

Motivated by the findings from our analysis of extant coral reefs, which revealed evidence of diverse trajectories of change that was dependent on genus (Fig. 2), but that was not related to family-level clades based on molecular trees [24], we asked whether the fossil record contained evidence of similar patterns. To answer this question, we focused on the geological record (6.8 to 0.125 Ma) during the late Miocene to late Pleistocene epochs. During the early Pliocene (5.3 to 3.6 Ma), mean sea-surface temperatures in the Caribbean were elevated 1–2°C — reflecting global mean temperatures 2–4°C higher than present [29] — and atmospheric *p*CO_2_ was as high as 400 ppmv [30]. This was followed by the colder and more thermally variable Pleistocene, which included intermittent glaciation.

To describe the dynamics of coral communities on fossil reefs, we drew on records extending from 0.125 to 6.8 Ma, which encompassed the separation of the Caribbean and Indo-Pacific biogeographic regions. Samples were extracted from outcrop exposures at 70 localities through four Miocene-Pleistocene sequences: Costa Rica [31]; Curacao [32]; Dominican Republic [33]; and Jamaica [34]. The collections comprised ∼6,528 specimens and 154 species, deposited at the US National Museum of Natural History (USNM), the University of Iowa (SUI) and the Natural History Museum in Basel, Switzerland (NMB). The specimens were identified to species using a standard set of morphological characters and character states, established in part by comparing morphological and molecular data and as detailed in the Neogene Biota of Tropical America (NMITA) taxonomic database [35]. Localities were grouped into faunules, which are defined as a set of lithologically similar localities from a small geographical area (usually <1 km) and restricted stratigraphic intervals (usually <20 m). The ages of faunules were assigned by integrating data from high-resolution chronostratigraphic methods that included nanofossil and planktonic foraminiferal biostratigraphy, paleomagnetics, and strontium isotope analyses [35], [36] and that generally ranged in accuracy from 0.5–2 Myr. The dataset consisted of counts of specimens belonging to species within each faunule, and is available on the NMITA website (http://nmita.iowa.uiowa.edu/index.htm).

Late Miocene and Pliocene coral reefs supported more diverse coral assemblages than extant coral reefs in the Caribbean, and their taxonomic composition overlapped at the genus level with contemporary Indo-Pacific coral communities. Approximately 80% of the>100 coral species became extinct during the Plio-Pleistocene, and over 60% of the species that now live in the modern Caribbean originated during that period [37]. The relative abundance of fossil genera for each faunule was assessed by dividing the number of specimens of a given genus by the total number of specimens collected within the faunule. The success of the fossil genera was examined by evaluating shifts in relative abundance over time using ordinary linear regression, and the slopes (% Myr^−1^) were used as a measure of relative success or failure (Table S2 in File S1). In a manner similar to that described above for contemporary reefs, coral genera on fossil reefs were defined as S-corals when their slopes were ≥0% Myr^−1^, and F-corals when their slopes were <0% Myr^−1^. Shifts in relative abundances also were scored based on whether genera subsequently became extinct in the Caribbean, and this analysis was used to provide insight into the evolutionary fates of S-corals and F-corals. To evaluate the relationship between overall abundance, trajectories of change, and extinction, the changes in relative abundances (% Myr^−1^) were plotted against the rank of relative dominance.

**Figure 3:**
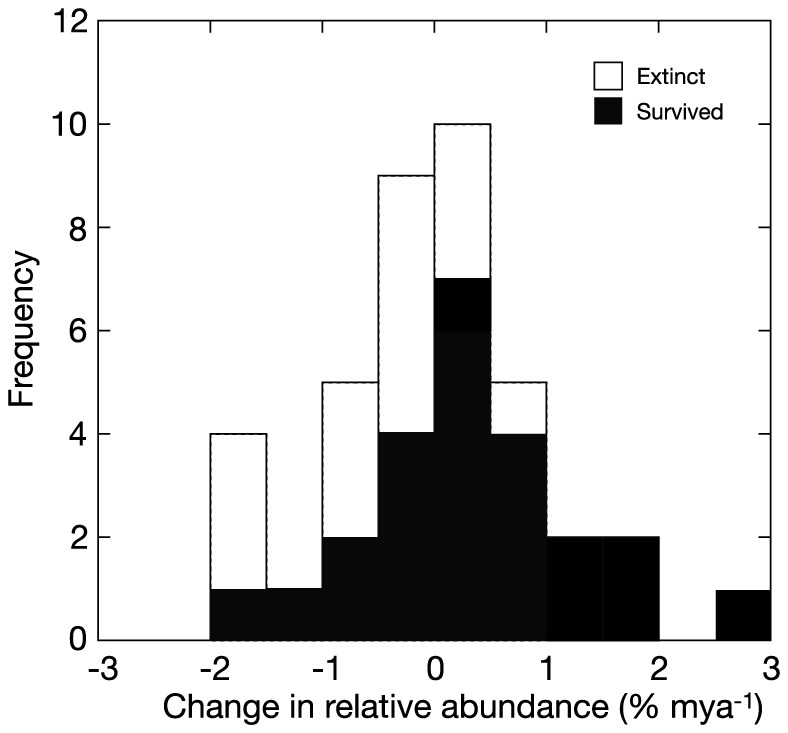
Changes in past coral communities as represented by the fossil record between 6.8 and 0.125 Ma. Changes in relative abundance of genera (% Myr^−1^) are shown. Histogram shows the changes in relative abundances for tropical taxa that became extinct (open bars) or survived (filled bars) in the Caribbean.

Fossil data revealed information on 39 coral genera, which showed normally distributed changes in relative abundances (*D* = 0.078, *n* = 39, *P* = 0.971), ranging from −1.758% Myr^−1^ (*Trachyphyllia*) to 2.650% Myr^−1^ (*Acropora*) (Fig. 3). Of the 39 genera, 15 became extinct and 10 of genera declined in relative abundance over the ∼6.7 Myr of the study. The probability of extinction tended to be dependent on the sign (i.e., ≥0 versus <0% Myr^−1^) of the slope of abundance on time (χ^2^ = 3.143, df = 1, *P* = 0.076). Analysis of the relationship between changes in relative abundance and dominance rank by genus (Fig. S2 in File S1) revealed no significant linear relationship between the two (*r* = 0.153, df = 37, *P* = 0.351), although more abundant taxa displayed larger shifts in relative abundances (both increases and decreases). Interestingly, coral families often were represented by some genera that increased in relative abundance and others that decreased. This suggests that the relative success of coral genera is independent of family affiliation. These results collectively are consistent with other work that focused on the fossil record and past extinction events [38], [39]. Extinction rates were higher in species with small colony sizes, but did not differ among species based on colony shape, corallite size, or reproductive mode [38]. However, during the Plio-Pleistocene faunal turnover in response to climate change, ecologically dominant and rare species appear equally susceptible to extinction, making S- and F- corals difficult to predict from these population characteristics [39].

In summary, our analysis of fossil communities demonstrated that coral genera responded differently to Plio-Pleistocene environmental perturbations (Table S3 in File S1). Approximately equal numbers of genera increased (51%) as decreased (49%) in relative abundance, and most extinctions (73%) affected coral genera that decreased in relative abundances. Our results reinforce the point that ecological dominance is not always linked to probability of extinction [39], [40]. However, dominance does affect the magnitude of changes in relative abundance, with dominant taxa prone to larger swings in relative abundance. While operating on a much longer time scale, the variable rates at which genera responded to environmental change in the fossil record provides a framework for interpreting differences in response of coral genera that are observed today. Assuming that changes in abundance over ecological time ultimately sum to create similar changes in abundance over geological time, then coral genera that are declining in abundance on contemporary reefs may be destined for extinction, while the relative abundances of genera that are currently dominant in coral communities are likely to change dramatically.

## Future

To build on our empirical support for the hypothesis that some corals have the potential to respond favorably to contemporary environmental changes, we developed a model of coral community composition to test the effects of specific coral traits on the trajectories of change in coral cover under increased thermal stress that is expected with future climate change. The traits that we examined included the classic life history traits of reproduction, growth, survivorship (mortality), maturation, and dispersal [41], the traits influencing coral-macroalgal interactions such as overgrowth resistance and herbivore recruitment, and thermal tolerance, which is a composite of physiological and morphological traits as well as symbiont composition. We focus on thermal stress because knowledge of its impact on coral demography allows explicit quantitative modeling. As thermal stress is only one aspect of current and future environmental change, we use a sensitivity analysis to explore the potential impacts of other components of change for which less is known (e.g., ocean acidification). The objective of our model was to evaluate the relative importance of coral life-history traits as predictors of coral cover under future climates [42]. We favored a trait-based model over a taxon-based model because our empirical evidence indicated that ecologically successful genera were scattered across multiple families. Thus, we reasoned that ecological function (i.e., traits) was a better indicator of ecological success than taxonomy, at least when success was gauged by changes in cover occurring over a time interval in which a variety of biotic and abiotic disturbances occurred unpredictably, as in the present analysis. As all of the traits considered affect overall population growth under disturbance conditions, and therefore persistence, there is no *a priori* reason to expect a particular trait to have more influence than another.

Mathematical models are used regularly to explore coral dynamics under general or specific climate change scenarios [e.g., 21,43,44]. Our goal was not to synthesize the existing models or to explicitly forecast the fate of any particular coral taxon. Instead, our intent was to build a generic model of the ecological dynamics of the full suite of different possible corals under expected future environments, where we consider each coral in isolation and use global sensitivity analysis (GSA [45]) to ask which biological processes and traits are most important in determining coral success. In other words, as a complement to previous analyses that investigate how a specified coral or coral reef community might respond to future disturbance, we sought to understand which coral traits mattered most to that response across reefs. Here, the GSA methodology lets us investigate those responses in the absence of knowledge on appropriate parameter values for the large number of coral species that exist.

### Model methods

**Figure 4:**
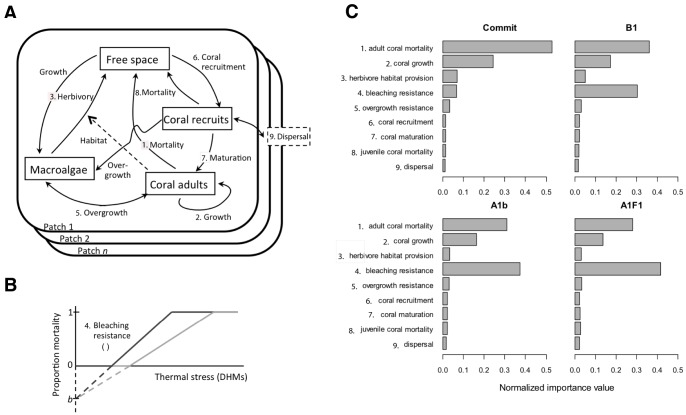
Model design. (A) The model tracks cover for coral recruits, coral adults, macroalgae, and free space across connected patches. Ecological processes (arrows; dashed line  =  indirect effect; solid lines  =  flow of energy, matter, organisms) govern changes in cover of state variables, numbered arrows correspond to processes evaluated through sensitivity analyses (C) for impact on community trajectories. Coral mortality subsumes shrinkage and baseline mortality. Parameter values are unboxed except for dispersal. (B) Bleaching resistance as revealed by the translation of degree heating months (DHMs) into mortality as shown by a line with an initial slope η. (C) Normalized importance values [66] for ecological processes for future climate change scenarios [40]. These GSA importance values represent the results of 1,000 simulations for each scenario, each with a unique set of randomly drawn parameter values, analyzed for which parameters were most influential for coral persistence.

Our model (Fig. 4A) is a stage-structured, continuous-time compartment model that tracks the proportion cover of coral (recruits and adults treated separately) and macroalgae in multiple patches connected by larval dispersal. In notation, let *R_i_, A_i_*, and *M_i_* represent the proportion cover of coral recruits, coral adults, and macroalgae in each patch *i* of *n* patches in total. Changes in each of these state variables are given by the differential equations: 
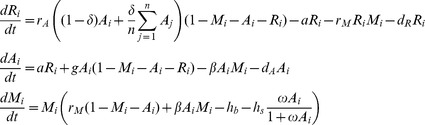
where *r_A_* represents coral recruitment (from reproduction by adults), *δ* is the proportion of dispersing larvae, *a* quantifies coral maturation, *g* represents adult coral growth, *d_R_* and *d_A_* represent recruit and adult coral loss of cover due to mortality and/or shrinkage, respectively; *r_M_* is algal growth, *h_b_* is baseline algal mortality (any herbivory and other algal senescence that would occur independent of coral density), *h_s_* is additional algal mortality from recruited herbivores (a rate with the same 1/time units as *h_b_*), *ω* scales the rate at which adult corals provide habitat for herbivores (units of 1/coral cover, such that the additional herbivory from corals is a saturating function that has a rate of saturation dictated by *ω*), and *β*≤*r_M_* is the rate at which algae overgrow adult corals depending on the degree of overgrowth resistance. Note our assumption here that the relative coral versus macroalgal cover affects grazing rate, as suggested in [46], [47]. We parameterized the model by using ranges of values based on comparable parameters used in recent models [19], [44] that encompassed empirical variation across coral taxa for ecological processes and biological traits (Table S4 in File S1). To focus the analysis on coral characteristics, we fixed parameters related to algal dynamics at single values that were determined similarly.

The model is driven by stochastic thermal anomalies with frequency and intensity drawn from the GFDL 2.1 climate model for 2051–2100 [48]. We compared model output under four IPCC scenarios: “Commit” with zero future greenhouse gas (GHG) emissions; B1 (550 ppm stabilization in 2100) representing large-scale future GHG emission reductions; A1B (700 ppm stabilization in 2100) and A1F1 describing future GHG emissions following “business-as-usual” trajectories, with greater emissions in A1F1. These climate scenarios were chosen to illustrate the range of possible future outcomes rather than to forecast a particular outcome. We applied annual, stochastic disturbances, using random draws from the degree heating months (DHMs) predicted for 2051–2100 [48] with normally distributed spatial variation among patches. Spatially heterogeneous thermal stresses allow for less impacted patches to supply larvae to more impacted patches (the “rescue effect” [49]), and permit the quantitative comparison of larval production and dispersal to reef persistence relative to other ecological processes. DHMs measure both the magnitude and duration that the temperature is above the average summer maximum, and are a predictor of bleaching events (η) [50]. Here, DHMs are translated into coral (both recruit and adult) mortality using a linear relationship, whose slope and zero-intercept quantify coral resistance to thermal anomalies (“bleaching resistance”, Fig. 4B). We ran simulations based on four of the locations from which the empirical data used in this study were obtained (Taiwan, Moorea, St. John, and Belize).

For each location and each climate scenario, we simulated dynamics for 1000 different collections of randomly chosen coral parameters. After discarding 50 y of transients, we recorded the average coral cover across all patches for 20 y. We then pooled simulations across locations and ran a global sensitivity analysis (GSA [45]) for each climate-change scenario to quantify the importance of each process and each trait in determining the coral cover on a multi-decadal scale. In brief, a GSA uses a ‘random forest’ of regression trees to create a predictive relationship between the randomly drawn coral parameters and the total coral cover under environmental disturbance. An ‘importance value’ is then calculated for each coral parameter by comparing the prediction accuracy of each tree with the parameter included versus excluded as a predictor. The importance value encompasses all effects of the parameter, including linear and non-linear effects and interactions with other parameters. Here, we used mean-squared error to quantify prediction accuracy, and normalized importance values to sum to 1 for each climate scenario. Processes and traits with large GSA scores strongly influenced long-term cover, and thus were considered influential in distinguishing coral taxa that function as strong ecological performers (S-corals) under the conditions specified.

We repeated the exercise for different simulation assumptions to determine the robustness of our results. In particular, we varied the number of patches, spatial variance in thermal stress, and the parameter range for coral recruitment. We investigated the recruit and adult cover sensitivity separately, and explored sensitivity with coral dynamics only (no macroalgae). The relative rankings presented are consistent across all of these tests as well as consistent across the four locations (consolidated in the results presented here).

As is inevitable with models, this model excludes more than it includes. For example, the model does not explicitly include other components of predicted future environments (e.g., increasing ocean acidity, increasing frequency and/or intensity hurricanes or typhoons, changing herbivory, nutrient runoff, over fishing, etc.), although we quantify the importance of the demographic processes that other environmental changes are expected to impact. For example, a high importance value for coral growth rates would suggest that total coral cover would be strongly influenced by decreases in growth rates from ocean acidification. The model also does not account for factors such as genetic adaptation of corals or their symbionts, competition or other interspecific interactions among multiple coral species on the same reef, hydrodynamical differences in susceptibility to disturbance based on relative position in the reef, or size-dependent demographics and disturbance susceptibility beyond our two stage classes (recruits and adults). These exclusions are necessary to keep the model tractable and transparent, but provide ample scope for future work.

### Model results

**Figure 5:**
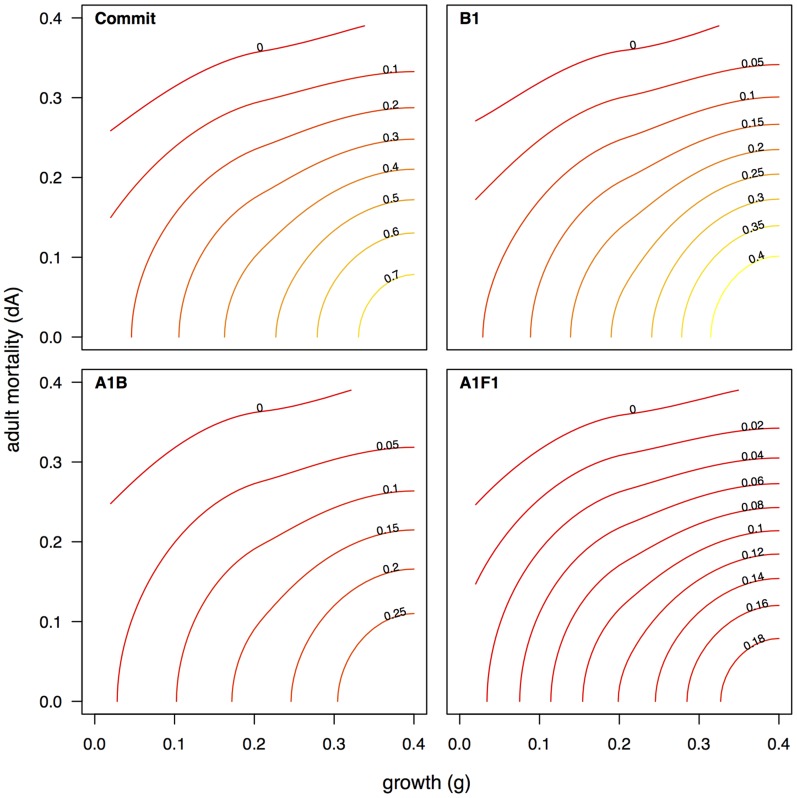
Contour plots for four IPCC climate-change scenarios (Commit, B1, A1b, and A1F1) showing the relationship between adult mortality (dA) versus growth rate (g) with the contours displaying the proportional coral cover. Contour plots were created by using a Loess smoother with average coral cover as the response variable, and g and dA as the predictors. Coral cover is greatest when growth rate is high and mortality is low. Increasingly severe bleaching mortality (i.e., as occurs in IPCC scenario A1F1) results in lower coral cover for a given combination of adult mortality and coral growth.

**Figure 6:**
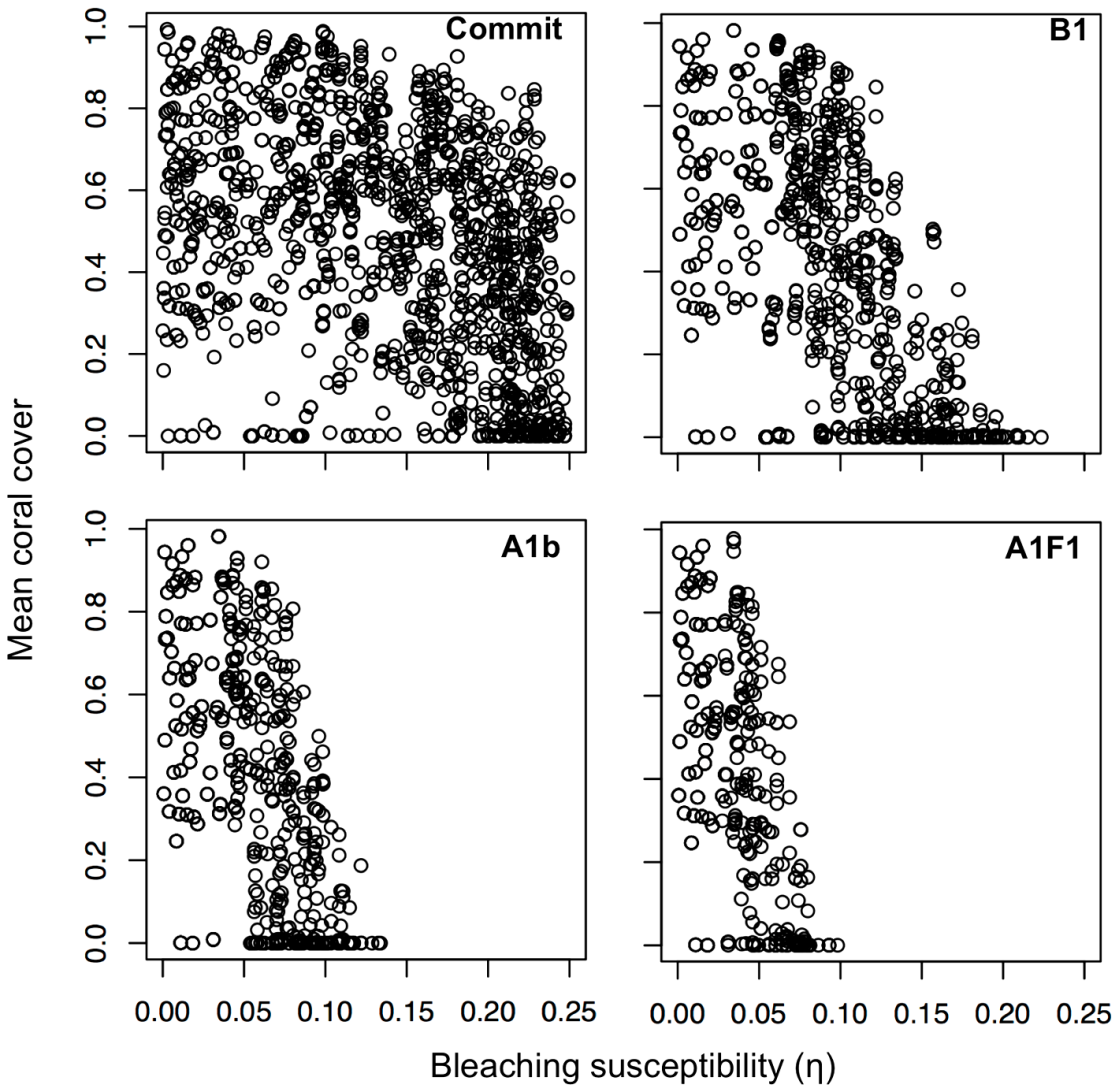
Mean coral cover versus bleaching susceptibility (η) for four IPCC climate-change scenarios (Commit, B1, A1b, and A1F1). Smaller (larger) values of η correspond to decreased (increased) susceptibility to bleaching mortality. In climate scenarios that predict more frequent and more intense thermal anomalies (e.g., A1b and A1F1), corals are only able to persist if their susceptibility to bleaching is below a threshold value. Points with zero coral cover are not shown.

Our GSA suggested that the most important ecological processes and biological traits favoring coral persistence (Fig. 4C) were adult coral mortality (i.e., mortality unrelated to bleaching) and adult coral growth (i.e., linear extension), with thermal tolerance becoming increasingly important under severe climate-change scenarios. Further analyses of the model results revealed a strong interaction between adult coral mortality and adult coral growth (Figs. 5, 6). Corals with rapid growth and moderate mortality are likely to persist, as are those with moderate growth and low mortality. Corals with slow growth and high adult mortality are unlikely to persist. The range of growth and mortality that favored persistence depended on the severity of the climate-change scenarios. In the most dire forecast, when temperature anomalies occurred nearly every year, corals needed to have at least two out of the three following traits: low adult mortality, rapid linear extension rates, and high tolerance of upward temperature excursions.

Of the possible two-way combinations between fast growth, low mortality, and high thermal tolerance, a coral having both low adult mortality and high thermal tolerance is most likely given life history trade-offs between growth and longevity, and the tendency for massive coral morphology to be associated with both slow growth and greater thermal tolerance [12]. Thermal tolerance, modeled here in terms of the minimum level of thermal stress before bleaching occurs and the rate of increase in mortality with increasing thermal stress (Fig. 4B), is a property that relates to a variety of characteristics, such as coral morphology [14], identity and flexibility of symbionts [51], genetic variation in thermal tolerance as it relates to adaptive capacity [52], and capacity for coral heterotrophy to supply energy and nutrients during warm thermal anomalies [53]. The importance of coral growth, adult mortality, and thermal tolerance to future coral reef growth parallels the findings from local sensitivity analyses and analogous results in other models that explore coral dynamics under expected future stress [e.g., 44,54–59]. The recurring identification of these three processes across a variety of models with different assumptions reinforces their biological significance.

While the most influential parameters: bleaching resistance, coral growth, and adult coral mortality are intuitively important, what is perhaps more surprising is that these three parameters clearly stand apart from others that relate to processes that have previously been identified as essential to future reef persistence. In particular, our model suggests that larval dispersal and recruitment, juvenile maturation, and competition with macroalgae (i.e., resistance to macroalgal overgrowth and provision of herbivore habitat), while always part of our modeled dynamics, are less influential in comparison with other processes in determining projected coral abundance. These results might seem to contradict those of other models in which recruitment dynamics and coral-macroalgal interactions are important to future coral persistence [19], [44], [55], [57], [58]. However, previous studies indicate that the presence versus absence of such dynamics is crucial, whereas our analysis addresses the separate question of whether or not the exact amount of recruitment and coral-macroalgal competition has a major role.

With respect to recruitment, previous studies indicate that larval exchange between multiple patches of coral experiencing different stress levels affects the outcome of coral population projections [56], [58]. Therefore, when evaluating coral community dynamics in models such as the one proposed here, it might be more important to incorporate the capacity for dispersal among reefs rather than the exact number of recruits involved in dispersal events. In addition, previous studies highlighting the importance of recruitment to the persistence of coral reefs note its greater influence on short-lived species than on long-lived species [59]. Therefore, if lower mortality and therefore a longer lifespan is central to persistence, then the set of surviving species is exactly the set for which recruitment plays less of a role. Analogously, a study of sea fans that used local sensitivity analysis noted that the observed low sensitivity to recruitment could be due, in part, to longevity [60]. In addition to shorter-lived species, recruitment and relative dispersal might be of greater importance for coral species with pulsed or stochastic recruitment [56], or when stress differs predictably among locations [56], [58].

With respect to coral-macroalgal competition, previous studies found coral cover was highly sensitive to competition with macroalgae, particularly where disturbances pushed coral cover close to an unstable threshold between coral-dominated and macroalgal-dominated states [58]. Therefore, parameters related to coral-macroalgal interactions might be particularly important under disturbance scenarios where switches between alternative stable states are likely. However, these parameters might be less influential relative to other processes outside this parameter space, reducing the overall importance, on average, across the broader disturbance regime and across the parameter ranges that drive the location of this threshold. The potential for alternative stable states in coral reefs is strongly debated, e.g., [61], [62], and might depend on the degree of anthropogenic degradation as well as on the processes that drive macroalgal growth capacity, which vary substantially within and across the Caribbean and Indo-Pacific [63], [64]. Elsewhere we demonstrate that the existence of alternative stable states can have the greatest effect in intermediate bleaching regimes, and change the relative importance of coral life history traits [65]. Lastly, our model makes several (common) simplifying assumptions that may have reduced the apparent importance of algal-coral competition and herbivore habitat provisioning. First, while our model aggregates algal somatic growth and reproduction (similar to, e.g., [19], [66]) more detailed simulation models that separate these processes tend to reveal stronger impacts of herbivory on coral-algal competition [67]. Second, skeletons of dead coral may continue to provide habitat for herbivores as they erode [68] and thus promote coral persistence in ways that our model does not capture.

We simulated coral reef community dynamics using climate model output from the fourth IPCC assessment report, released in 2007. Sea surface temperature projections are available from the fifth IPCC assessment report, released in 2013. However, large increases in mean and maximum sea surface temperature in AR5 relative to AR4 caused decreases in simulated coral cover to under 10% in all model run and extirpation in>90% (Fig. S3 in File S1). Despite these major quantitative differences, the AR4 and AR5 model output generate the same qualitative life- history trait importance values (Fig. S4 in File S1). Thus, we chose to illustrate our qualitative results using the AR4 model output because the variance is clearer. The pessimistic nature of the AR5 simulation results should be contextualized in the fact that our model did not consider coral and symbiont acclimation and adaptation, which could increase coral cover or persistence [43], [58], [69]. Moreover, the AR4 model output may be more accurate than AR5 for specific locations or time periods [70].

Overall, our analysis suggests that future reefs will be populated at low densities by scleractinian corals with at least two of the following traits: high thermal tolerance, fast growth, and low adult mortality (Figs. 4C, 5, 6). The interactive dependence of reef persistence on bleaching resistance, growth, and mortality suggests potential synergistic interaction of future thermal stress with ocean acidification (which affects coral calcification and therefore growth) and temperature-triggered diseases (which affect coral mortality). It is likely that coral species will differ in their susceptibility to the effects of ocean acidification [71] and diseases [72] as much as they will to future bleaching. Therefore, a critical challenge for future research will be to combine the GSA approach used here with models that incorporate multiple interacting impacts (e.g., [55]) to provide an understanding of characteristics most relevant to the persistence of corals under multiple types of disturbances.

### Synthesis and Conclusions

We asked whether empirical data from the US Virgin Islands and Belize in the Caribbean, and Moorea, Taiwan, Hawaii, the Great Barrier Reef, and Kenya in the Indo-Pacific, supported a diversity of outcomes for the changing cover of coral genera over the coming century. Our results confirm that coral genera have experienced widespread declines in abundance since the 1980s, with the changes more acute in the Caribbean than in the Indo-Pacific. The results also reveal that some coral genera have maintained or increased their relative and absolute abundance in the study locations over this same period. In our two Caribbean locations, more genera decreased (n = 11) than increased (n = 5) in absolute abundance, but almost equal numbers increased (n = 21) and decreased (n = 20) in the five Indo-Pacific locations. These results suggest that the future of scleractinian corals over the current century will likely include the persistence and perhaps increased abundances of some coral genera, even while others become less common. These results highlight the strong likelihood that changes in the generic-level composition of coral communities will occur in coming decades [73]–[75]. Changes in generic-level coral-community structure likely will affect the capacity of coral reefs to deliver the goods and services for which they have been well known. The coral genera that will populate future reefs will probably be the strong ecological performers on contemporary reefs, although this conclusion must be tempered by the reality that past performance is an imperfect indicator of future success [76], particularly as the time horizon lengthens. Although the fossil record suggests that community dominance is not a good indicator of long-term persistence, nevertheless long-term increases in relative abundance are generally characteristic of coral genera that persist rather than go extinct, whereas long-term declines tend to culminate in extinction. Translating changes in community structure that have occurred over millennia to implications for coral dynamics over ecological time is problematic, however the fossil record offers a pessimistic prognosis for coral genera that are not currently achieving ecological success (i.e., F-domain corals).

We used modeling to identify traits associated with S-domain corals under a limited subset of future climatic conditions, and this effort highlighted the importance of thermal resistance, rapid growth of reproductively mature corals, and coral longevity. Corals possessing at least two of these three traits are most likely to dominate coral-reef communities in coming decades. It would be desirable to screen extant coral genera for these traits and use the results to couple taxonomic data to the model predictions, but the empirical data to fuel this effort are still sparse [16], [77]. While considerable knowledge exists for a small number of coral taxa – like Indo-Pacific *Acropora*, *Stylophora*, and *Pocillopora*, and Caribbean *Acropora*, *Orbicella*, and *Madracis* – relatively little is known about the functional biology of taxa that are emerging as S-domain corals (Table S3 in File S1). Describing the functional biology of these genera and elucidating how functional traits scale up to determine critical demographic properties, like the intrinsic rate of population increase, must be a research priority for future studies. Such efforts will be critical to guide future empirical work and to predict the functionality of future coral reef ecosystems.

## Supporting Information

File S1 **Combined Supporting Information file.**
(DOCX)
