# Supplementary material for: Persistence and Change in Community Composition of Reef Corals through Present, Past, and Future Climates
Source: PLoS One. 2014 Oct 1;9(10):e107525. doi: 10.1371/journal.pone.0107525 (PMC4182679; doi:10.1371/journal.pone.0107525)
Supplement: File S1 — Combined Supporting Information file. (DOCX) [file pone.0107525.s001.docx]

SUPPORTING INFORMATION

**Persistence and change in community composition of reef corals through present, past and future climates**

Peter J. Edmunds, Mehdi Adjeroud, Marissa L. Baskett, Iliana B. Baums, Ann F. Budd, Robert C. Carpenter, Nicholas S. Fabina, Tung-Yung Fan, Erik C. Franklin, Kevin Gross, Xueying Han, Lianne Jacobson, James S. Klaus, Tim R. McClanahan, Jennifer K. O’Leary, Madelaine J.H. van Oppen, Xavier Pochon, Hollie M. Putnam, Tyler B. Smith, Michael Stat, Hugh Sweatman, Robert van Woesik, Ruth D. Gates

Table S1. Summary of long-term study sites used in this study. 1) Eight sites scattered among three habitats: a deep reef (14 m, Tektite) dominated by *Orbicella annularis* (censused 1987-2012), a shallower reef (9 m, Yawzi Point) dominated by *O. annularis* (censused 1987-2012), and a fringing reef at 7-9 m depth along which 6 sites are scattered (censused 1992-2012). All sites have been censused annually, and lie along < 10 km of shore. For the present analysis, the data are separated by habitat (Yawi Point, Tektite, and the pooled sites). 2) Ten sites (each with ten, 10 m permanent transects) in the US Virgin Islands between at 7-13 m depth were surveyed annually from 2001-2012. St. Croix included sites dominated by *O.* *annularis* at Buck Island, Cane Bay, and Sprat Hole, and a mixed coral community at Eagle Ray, Jack’s Bay, and Salt River West; St. John included a mixed coral community on a fringing reef in Fish Bay; St. Thomas included a mixed coral community on fringing reefs at Coculus Rock, Botany Bay, and Magens Bay. 3) Eight patch reefs (= sites) scattered along the southeast edge of Glover’s Reef Atoll, all within 0.5-2.0 m depth. 4) Six sites surrounding Moorea (2 sites shore^-1^), that sample the outer reef (10-m depth) and the fringing reef (5-6 m depth) along ~50 km of shore. These sites have been sampled annually since 2005 and for the present study are pooled among sites and shores. 5) One site at the Tiahura Sector of the outer reef (10-12 m depth) on the north shore of Moorea. 6) Four sites were surveyed annually at 3-5 m depth near Kahe Point, on the leeward shore of Oahu, Hawaii. 7) Four sites were surveyed annually from 2007-2010 along 3 permanent, 15-meter transects at 1-4 m depth on the reef flat. 8) Twelve sites at 0.5-2.0 m depth. 9) Three sites at 6-10 m depth were surveyed on each of 47 reefs, annually until 2005, then in alternate years. For the present analysis, data were aggregated to three positions across the GBR lagoon: inshore (11 reefs), midshelf (18 reefs) and outer shelf (18 reefs). Refer to references for further details of these sites.

Table S1

| Location | Coordinates | Duration | Method | Habitat | Source | Reference |
| --- | --- | --- | --- | --- | --- | --- |
| **CARIBBEAN** |  |  |  |  |  |  |
| 1. St. John, USVI | 18° 18’ N, 64° 43’ W | 1987-2012 | Photoquadrats | Fringing reef | P.J. Edmunds | 78 |
| 2. St. Thomas & St. Croix, USVI | 18° 00 ‘ N, 64° 48‘W | 2001-2012 | Video transects | Fringing reef | T.B. Smith | 79 |
| 3. Belize | 16° 45’N, 87° 48’ W | 1996-2010 | Line intercept transects | Back reef atoll | T.R. McClanahan | 80 |
| **INDO-PACIFIC** |  |  |  |  |  |  |
| 4. Moorea | 17° 28’ S, 149° 50’ W | 2005-2012 | Photoquadrats | Outer reef and fringing reef | MCR-LTER | 81 |
| 5. Moorea | 17° 30’S, 149° 50’ W | 1991-2006 | Point intercept transects | Outer reef | CRIOBE/M. Adjeroud | 4 |
| 6. Oahu, Hawaii | 21° 21’ N, 158° 08’ W | 1981-2005 | Photoquadrats | Fringing reef | S.L. Coles, E.K. Brown | 82 |
| 7. Taiwan | 21° 57’ N, 120° 45’ E | 2007-2010 | Photoquadrats | Fringing reef | T.Y. Fan |  |
| 8. Kenya | 4° 03’ S, 39° 40’ E | 1987-2010 | Line intercept transects | Subtidal reef flat | T.R. McClanahan | 83 |
| 9. Great Barrier Reef | 14° 33’ S, 145° 34’ E  to  23° 54’ S, 152° 24’ E | 1995-2013 | Photoquadrats | NE reef slopes | H. Sweatman | 84, 85 |

Table S2.

**Recent Data.** Summary of change in coral cover over time on both absolute and relative scales for the genera in the data compilation from sites in the Caribbean and Indo-Pacific. Not all genera are included in this analysis, as some were too rare to allow meaningful trajectories of change to be calculated. Start and End of time series shown together with the number of years censused (N), together with the slope of the regression of cover (absolute and relative) on time (% y^-1^), the standard error of this value, and the regression statistics (F, df, and *P*-value). Significant values in bold. Sites as described in Table S1.

| *Family* | Genus | Region | Site | Start Y | End Y | N | Type | Slope % y^-1^ | SE | Regression statistics |
| --- | --- | --- | --- | --- | --- | --- | --- | --- | --- | --- |
| Merulinidae | *Orbicella* | Caribbean | St. John-PRS | 1992 | 2012 | 21 | Absolute | -0.0081 | 0.0120 | F = 0.445, df = 1,19, P = 0.513 |
|  |  |  |  |  |  |  | Relative | -0.0940 | 0.2790 | F = 0.113, df = 1,19, P = 0.740 |
| Montastraeidae | *Montastraea* | Caribbean | St. John-PRS | 1992 | 2012 | 21 | Absolute | -0.0076 | 0.0040 | F = 3.273, df = 1,19, P = 0.086 |
|  |  |  |  |  |  |  | Relative | -0.1617 | 0.1090 | F = 2.193, df = 1,19, P = 0.155 |
| Agaricidae | *Agaricia* | Caribbean | St. John-PRS | 1992 | 2012 | 21 | Absolute | **-0.0162** | **0.0060** | **F = 7.375, df = 1,19, P = 0.014** |
|  |  |  |  |  |  |  | Relative | **-0.4169** | **0.1480** | **F = 7.965, df = 1,19, P = 0.011** |
| Meandrinidae | *Dichocoenia* | Caribbean | St. John-PRS | 1992 | 2012 | 21 | Absolute | 0.0024 | 0.0030 | F = 0.851, df = 1,19, P = 0.368 |
|  |  |  |  |  |  |  | Relative | 0.0664 | 0.0700 | F = 0.910, df = 1,19, P = 0.352 |
| Mussidae | *Diploria* | Caribbean | St. John-PRS | 1992 | 2012 | 21 | Absolute | -0.0003 | 0.0030 | F = 0.005, df = 1,19, P = 0.943 |
|  |  |  |  |  |  |  | Relative | 0.0277 | 0.0910 | F = 0.093, df = 1,19, P = 0.764 |
| Meandrinidae | *Meandrina* | Caribbean | St. John-PRS | 1992 | 2012 | 21 | Absolute | 0.0007 | 0.0030 | F = 0.084, df = 1,19, P = 0.775 |
|  |  |  |  |  |  |  | Relative | 0.0201 | 0.0620 | F = 0.105, df = 1,19, P = 0.750 |
| Poritidae | *Porites* | Caribbean | St. John-PRS | 1992 | 2012 | 21 | Absolute | **0.0196** | **0.0050** | **F = 17.985, df = 1,19, P < 0.001** |
|  |  |  |  |  |  |  | Relative | **0.6542** | **0.1570** | **F = 17.435, df = 1,19, P = 0.005** |
| Astrocoeniidae | *Stephanocoenia* | Caribbean | St. John-PRS | 1992 | 2012 | 21 | Absolute | -0.0029 | 0.0010 | F = 4.199, df = 1,19, P = 0.055 |
|  |  |  |  |  |  |  | Relative | **-0.0781** | **0.0370** | **F = 4.374, df = 1,19, P = 0.050** |
| Siderastreidae | *Siderastrea* | Caribbean | St. John-PRS | 1992 | 2012 | 21 | Absolute | **-0.0284** | **0.0090** | **F = 9.609, df = 1,19, P = 0.006** |
|  |  |  |  |  |  |  | Relative | **-0.6000** | **0.2360** | **F = 6.450, df =1,19, P = 0.020** |
| Merulinidae | *Orbicella* | Caribbean | St. John-Yawzi | 1987 | 2012 | 9 | Absolute | **-1.4233** | **0.1530** | **F = 86.683, df = 1,7, P < 0.001** |
|  |  |  |  |  |  |  | Relative | **-0.3820** | **0.1610** | **F = 5.620, df = 1,7, P = 0.050** |
| Montastraeidae | *Montastraea* | Caribbean | St. John-Yawzi | 1987 | 2012 | 9 | Absolute | -0.0001 | 0.0060 | F < 0.001, df = 1,7, P = 0.992 |
|  |  |  |  |  |  |  | Relative | 0.0550 | 0.1030 | F = 0.290, df = 1,7, P = 0.607 |
| Poritidae | *Porites* | Caribbean | St. John-Yawzi | 1987 | 2012 | 9 | Absolute | -0.0087 | 0.0110 | F = 0.706, df = 1,7, P = 0.429 |
|  |  |  |  |  |  |  | Relative | 0.2544 | **0.0850** | **F = 9.048, df = 1,7, P = 0.020** |
| Siderastreidae | *Siderastrea* | Caribbean | St. John-Yawzi | 1987 | 2012 | 9 | Absolute | -0.0034 | 0.0050 | F = 0.575, df = 1,7, P = 0.473 |
|  |  |  |  |  |  |  | Relative | 0.0572 | 0.0250 | F = 5.313, df = 1,7, P = 0.055 |
| Merulinidae | *Orbicella* | Caribbean | St. John-Tektite | 1987 | 2012 | 9 | Absolute | -0.1551 | 0.3150 | F = 0.244, df = 1,7, P = 0.636 |
|  |  |  |  |  |  |  | Relative | 0.0770 | 0.0990 | F = 0.602, df = 1,7, P = 0.463 |
| Montastraeidae | *Montastraea* | Caribbean | St. John-Tektite | 1987 | 2012 | 9 | Absolute | 0.0017 | 0.0080 | F = 0.051, df = 1,7, P = 0.827 |
|  |  |  |  |  |  |  | Relative | 0.0077 | 0.0260 | F = 0.087, df = 1,7, P = 0.777 |
| Agaricidae | *Agaricia* | Caribbean | St. John-Tektite | 1987 | 2012 | 9 | Absolute | -0.0243 | 0.0120 | F = 4.104, df = 1,7, P = 0.082 |
|  |  |  |  |  |  |  | Relative | **-0.0647** | **0.0240** | **F = 7.057, df = 1,7, P = 0.033** |
| Mussidae | *Colpophyllia* | Caribbean | St. John-Tektite | 1987 | 2012 | 9 | Absolute | -0.0518 | 0.0220 | F = 5.390, df = 1,7, P = 0.053 |
|  |  |  |  |  |  |  | Relative | **-0.1438** | **0.0510** | **F = 8.069, df = 1,7, P = 0.025** |
| Mussidae | *Diploria* | Caribbean | St. John-Tektite | 1987 | 2012 | 9 | Absolute | -0.0200 | 0.0160 | F = 1.594, df = 1,7, P = 0.247 |
|  |  |  |  |  |  |  | Relative | -0.0511 | 0.0410 | F = 1.566, df = 1,7, P = 0.251 |
| Poritidae | *Porites* | Caribbean | St. John-Tektite | 1987 | 2012 | 9 | Absolute | 0.0491 | 0.0280 | F = 3.027, df = 1,7, P = 0.125 |
|  |  |  |  |  |  |  | Relative | **0.1773** | **0.0640** | **F = 7.584, df = 1,7, P = 0.028** |
| Siderastreidae | *Siderastrea* | Caribbean | St. John-Tektite | 1987 | 2012 | 9 | Absolute | **-0.0254** | **0.0060** | **F = 19.521. df = 1,7, P = 0.003** |
|  |  |  |  |  |  |  | Relative | **-0.0647** | **0.0260** | **F = 6.160, df = 1,7, P = 0.042** |
| Acroporidae | *Acropora* | Indo-Pacific | Moorea-Fringe | 2005 | 2012 | 8 | Absolute | **-0.0283** | **0.0080** | **F = 12.5782, df = 1,7, P = 0.008** |
|  |  |  |  |  |  |  | Relative | -0.0351 | 0.0490 | F = 0.524, df = 1,7, P = 0.497 |
| Fungidae | *Fungia* | Indo-Pacific | Moorea Fringe | 2005 | 2012 | 8 | Absolute | -0.0042 | 0.0020 | F = 5.097, df = 1,7, P = 0.065 |
|  |  |  |  |  |  |  | Relative | -0.0117 | 0.0050 | F = 4.614, df = 1,7, P = 0.075 |
| Acroporidae | *Montipora* | Indo-Pacific | Moorea Fringe | 2005 | 2012 | 8 | Absolute | **-0.3799** | **0.0770** | **F = 24.654, df = 1,7, P = 0.003** |
|  |  |  |  |  |  |  | Relative | **-0.6438** | **0.2260** | **F = 8.123, df = 1,7, P = 0.029** |
| Agaricidae | *Pavona* | Indo-Pacific | Moorea Fringe | 2005 | 2012 | 8 | Absolute | **-0.1681** | **0.0550** | **F = 9.446, df = 1,7, P = 0.022** |
|  |  |  |  |  |  |  | Relative | 0.0839 | 0.2520 | F = 0.111, df = 1,7, P = 0.751 |
| Pocilloporidae | *Pocillopora* | Indo-Pacific | Moorea Fringe | 2005 | 2012 | 8 | Absolute | **-0.0274** | **0.0110** | **F = 6.830, df = 1,7, P = 0.0399** |
|  |  |  |  |  |  |  | Relative | **-0.0904** | **0.0350** | **F = 6.784, df = 1,7, P = 0.035** |
| Poritidae | *Porites* | Indo-Pacific | Moorea Fringe | 2005 | 2012 | 8 | Absolute | -0.5671 | 0.4270 | F = 1.766, df = 1,7, P = 0.232 |
|  |  |  |  |  |  |  | Relative | 0.6402 | 0.4340 | F = 2.176, df = 1,7, P = 0.191 |
| Acroporidae | *Acropora* | Indo-Pacific | Moorea 10 m | 2005 | 2012 | 8 | Absolute | **-1.4164** | **0.2560** | **F = 30.511, df = 1,7, P = 0.002** |
|  |  |  |  |  |  |  | Relative | **-3.4264** | **0.6100** | **F = 31.426, df = 1,7, P = 0.001** |
| Acroporidae | *Astreopora* | Indo-Pacific | Moorea 10 m | 2005 | 2012 | 8 | Absolute | **-0.0545** | **0.0130** | **F = 16.450, df = 1,7, P = 0.007** |
|  |  |  |  |  |  |  | Relative | 0.0056 | 0.1180 | F = 0.002, df = 1,7, P = 0.964 |
| Fungidae | *Fungia* | Indo-Pacific | Moorea 10 m | 2005 | 2012 | 8 | Absolute | **-0.0487** | **0.0090** | **F = 28.428, df = 1,7, P = 0.002** |
|  |  |  |  |  |  |  | Relative | **-0.1276** | **0.0160** | **F = 62.601, df = 1,7, P = 0.002** |
| Incertae sedis | *Leptastrea* | Indo-Pacific | Moorea 10 m | 2005 | 2012 | 8 | Absolute | **-0.1457** | **0.0250** | **F = 32.992, df = 1,7, P = 0.001** |
|  |  |  |  |  |  |  | Relative | 0.2692 | 0.1440 | F = 3.490, df = 1,7, P = 0.111 |
| Merulinidae | *Phymastrea* | Indo-Pacific | Moorea 10 m | 2005 | 2012 | 8 | Absolute | -0.0421 | 0.0190 | F = 4.753, df = 1,7, P = 0.072 |
|  |  |  |  |  |  |  | Relative | **0.9369** | **0.2200** | **F = 18.127, df = 1,7, P = 0.005** |
| Acroporidae | *Montipora* | Indo-Pacific | Moorea 10 m | 2005 | 2012 | 8 | Absolute | **-0.4541** | **0.0540** | **F = 71.117, df = 1,7, P < 0.001** |
|  |  |  |  |  |  |  | Relative | 0.8099 | 0.4120 | F = 3.874, df = 1,7, P < 0.097 |
| Agaricidae | *Pavona* | Indo-Pacific | Moorea 10 m | 2005 | 2012 | 8 | Absolute | **0.1011** | **0.0190** | **F = 27.939, df = 1,7, P = 0.002** |
|  |  |  |  |  |  |  | Relative | 0.1695 | 0.1010 | F = 2.798, df = 1,7, P = 0.145 |
| Pocillporidae | *Pocillopora* | Indo-Pacific | Moorea 10 m | 2005 | 2012 | 8 | Absolute | **-3.0245** | **0.3990** | **F = 57.460, df = 1,7, P < 0.001** |
|  |  |  |  |  |  |  | Relative | -0.9852 | 0.9890 | F = 0.993. df = 1,7, P = 0.358 |
| Poritidae | *Porites* | Indo-Pacific | Moorea 10 m | 2005 | 2012 | 8 | Absolute | **-0.7520** | **0.0870** | **F = 74.654, df = 1,7, P < 0.001** |
|  |  |  |  |  |  |  | Relative | **1.9813** | **0.4400** | **F = 20.258, df = 1,7, P = 0.004** |
| Psammocoridae | *Psammocora* | Indo-Pacific | Moorea 10 m | 2005 | 2012 | 8 | Absolute | -0.0006 | 0.0070 | F = 0.006, df = 1,7, P = 0.941 |
|  |  |  |  |  |  |  | Relative | 0.3013 | **0.1290** | **F = 5.487, df = 1,7, P = 0.058** |
| Acroporidae | *Acropora* | Indo-Pacific | Moorea 17 m | 2005 | 2012 | 8 | Absolute | **-1.3344** | **0.3830** | **F = 22.302, df = 1,7, P = 0.003** |
|  |  |  |  |  |  |  | Relative | **-3.8074** | **0.8240** | **F = 21.344, df = 1,7, P = 0.004** |
| Acroporidae | *Astreopora* | Indo-Pacific | Moorea 17 m | 2005 | 2012 | 8 | Absolute | **-0.0226** | **0.0070** | **F = 11.056, df = 1,7, P = 0.016** |
|  |  |  |  |  |  |  | Relative | -0.0431 | 0.0230 | F = 3.268, df = 1,7, P = 0.121 |
| Fungidae | *Fungia* | Indo-Pacific | Moorea 17 m | 2005 | 2012 | 8 | Absolute | **-0.2614** | **0.0560** | **F = 21.699, df = 1,7, P = 0.004** |
|  |  |  |  |  |  |  | Relative | -0.7063 | **0.1530** | **F = 21.231, df = 1,7, P = 0.004** |
| Incertae sedis | *Leptastrea* | Indo-Pacific | Moorea 17 m | 2005 | 2012 | 8 | Absolute | **-0.2026** | **0.0430** | **F = 22.673, df = 1,7, P = 0.003** |
|  |  |  |  |  |  |  | Relative | **-0.4375** | **0.1060** | **F = 16.978, df = 1,7, P = 0.006** |
| Merulinidae | *Phymastrea* | Indo-Pacific | Moorea 17 m | 2005 | 2012 | 8 | Absolute | -0.0421 | 0.0240 | F = 3.053, df = 1,7, P = 0.131 |
|  |  |  |  |  |  |  | Relative | **1.2857** | **0.1580** | **F = 66.265, df = 1,7, P < 0.001** |
| Acroporidae | *Montipora* | Indo-Pacific | Moorea 17 m | 2005 | 2012 | 8 | Absolute | **-0.1765** | **0.0400** | **F = 19.438, df = 1,7, P = 0.005** |
|  |  |  |  |  |  |  | Relative | 0.3226 | 0.2730 | F = 5.389, df = 1,7, P = 0.283 |
| Agaricidae | *Pavona* | Indo-Pacific | Moorea 17 m | 2005 | 2012 | 8 | Absolute | **-0.1382** | **0.0370** | **F = 13.839, df = 1,7, P = 0.010** |
|  |  |  |  |  |  |  | Relative | 0.4296 | 0.2730 | F = 2.468, df = 1,7, P = 0.167 |
| Pocilloporidae | *Pocillopora* | Indo-Pacific | Moorea 17 m | 2005 | 2012 | 8 | Absolute | -2.0263 | **0.4160** | **F = 23.770, df = 1,7, P = 0.003** |
|  |  |  |  |  |  |  | Relative | -2.0529 | 1.2840 | F = 2.556, df = 1,7, P = 0.161 |
| Poritidae | *Porites* | Indo-Pacific | Moorea 17 m | 2005 | 2012 | 8 | Absolute | **-1.0819** | **0.1990** | **F = 29.658, df = 1,7, P = 0.002** |
|  |  |  |  |  |  |  | Relative | 4.7510 | 2.2960 | F = 4.751, df = 1,7, P = 0.084 |
| Psammocoridae | *Psammocora* | Indo-Pacific | Moorea 17 m | 2005 | 2012 | 8 | Absolute | -0.0593 | 0.0270 | F = 4.814, df = 1,7, P = 0.071 |
|  |  |  |  |  |  |  | Relative | 0.2150 | 2.2960 | F = 1.273, df = 1,7, P = 0.302 |
| Incertae sedis | *Leptastrea* | Indo-Pacific | Hawaii - Kahe | 1981 | 2005 | 25 | Absolute | -0.0010 | 0.0020 | F = 0.178, df = 1,23, P = 0.677 |
|  |  |  |  |  |  |  | Relative | -0.0007 | 0.0150 | F = 0.002, df = 1,23, P = 0.962 |
| Acroporidae | *Montipora* | Indo-Pacific | Hawaii – Kahe | 1981 | 2005 | 25 | Absolute | **0.0428** | **0.0150** | **F = 7.742, df = 1,23, P = 0.011** |
|  |  |  |  |  |  |  | Relative | **0.3365** | **0.1000** | **F = 11.282, df = 1,23, P = 0.003** |
| Pocilloporidae | *Pocillopora* | Indo-Pacific | Hawaii – Kahe | 1981 | 2005 | 25 | Absolute | -0.0269 | 0.0360 | F = 0.5748, df = 1,23, P = 0.456 |
|  |  |  |  |  |  |  | Relative | 0.0282 | 0.1460 | F = 0.0377, df = 1,23, P = 0.848 |
| Poritidae | *Porites* | Indo-Pacific | Hawaii – Kahe | 1981 | 2005 | 25 | Absolute | **-0.1547** | **0.0410** | **F = 13.933, df = 1,23, P = 0.001** |
|  |  |  |  |  |  |  | Relative | **-0.3640** | **0.1060** | **F = 11.819, df = 1,23, P = 0.002** |
| Lobophyllidae | *Acanthastrea* | Indo-Pacific | Tiahura slope | 1991 | 2006 | 16 | Absolute | -0.0600 | 0.0030 | F = 3.000, df = 1,14, P = 0.105 |
|  |  |  |  |  |  |  | Relative | -0.0110 | 0.0060 | F = 3.000, df = 1,14, P = 0.105 |
| Acroporidae | *Acropora* | Indo-Pacific | Tiahura slope | 1991 | 2006 | 16 | Absolute | **0.5350** | **0.1460** | **F = 13.421, df = 1,14, P = 0.003** |
|  |  |  |  |  |  |  | Relative | **0.7530** | **0.2210** | **F = 11.664, df = 1,14, P = 0.004** |
| Fungidae | *Fungia* | Indo-Pacific | Tiahura slope | 1991 | 2006 | 16 | Absolute | 0.0230 | 0.0200 | F = 1.339, df = 1,14, P = 0.267 |
|  |  |  |  |  |  |  | Relative | -0.0070 | 0.0480 | F = 0.024, df = 1,14, P = 0.880 |
| Fungidae | *Herpolitha* | Indo-Pacific | Tiahura slope | 1991 | 2006 | 16 | Absolute | 0.0330 | 0.0140 | F = 5.379, df = 1,14, P = 0.036 |
|  |  |  |  |  |  |  | Relative | 0.0680 | 0.0330 | F = 4.309, df = 1,14, P = 0.057 |
| Incertae sedis | *Leptastrea* | Indo-Pacific | Tiahura slope | 1991 | 2006 | 16 | Absolute | 0.0240 | 0.0260 | F = 0.800, df = 1,14, P = 0.399 |
|  |  |  |  |  |  |  | Relative | -0.0900 | 0.0800 | F = 1.277, df = 1,14, P = 0.277 |
| Merulinidae | *Phymastrea* | Indo-Pacific | Tiahura slope | 1991 | 2006 | 16 | Absolute | 0.0060 | 0.0180 | F = 0.103, df = 1,14, P = 0.753 |
|  |  |  |  |  |  |  | Relative | -0.0310 | 0.0380 | F = 0.664, df = 1,14, P = 0.429 |
| Acroporidae | *Montipora* | Indo-Pacific | Tiahura slope | 1991 | 2006 | 16 | Absolute | -0.0550 | 0.0890 | F = 0.376, df = 1,14, P = 0.549 |
|  |  |  |  |  |  |  | Relative | -0.3870 | 0.1570 | F = 6.109, df = 1,14, P = 0.027 |
| Agaricidae | *Pavona* | Indo-Pacific | Tiahura slope | 1991 | 2006 | 16 | Absolute | 0.0720 | 0.0380 | F = 3.570, df = 1,14, P = 0.080 |
|  |  |  |  |  |  |  | Relative | 0.0490 | 0.0940 | F = 0.267, df = 1,14, P = 0.624 |
| Pocilloporidae | *Pocillopora* | Indo-Pacific | Tiahura slope | 1991 | 2006 | 16 | Absolute | 0.0860 | 0.2000 | F = 0.188, df = 1,14, P = 0.672 |
|  |  |  |  |  |  |  | Relative | **-1.0620** | **0.2370** | **F = 20.045, df = 1,14, P < 0.001** |
| Poritidae | *Porites* | Indo-Pacific | Tiahura slope | 1991 | 2006 | 16 | Absolute | **0.5960** | **0.0550** | **F = 117.438, df = 1,14, P < 0.001** |
|  |  |  |  |  |  |  | Relative | **0.6020** | **0.1890** | **F = 10.158, df = 1,14, P = 0.007** |
| Acroporidae | *Acropora* | Indo-Pacific | Taiwan outlet | 2007 | 2010 | 4 | Absolute | -0.4833 | 0.8139 | F = 1.763, df =1,2, P = 0.316 |
|  |  |  |  |  |  |  | Relative | -1.6359 | 2.2099 | F = 2.740, df =1,2, P = 0.240 |
| Coscinaraeidae | *Coscinaraea* | Indo-Pacific | Taiwan outlet | 2007 | 2010 | 4 | Absolute | 0.0126 | 0.0743 | F = 0.143, df =1,2, P = 0.742 |
|  |  |  |  |  |  |  | Relative | 0.0314 | 0.1856 | F = 0.143, df =1,2, P = 0.742 |
| Merulinidae | *Cyphastrea* | Indo-Pacific | Taiwan outlet | 2007 | 2010 | 4 | Absolute | 0.0297 | 0.9122 | F = 0.041, df =1,2, P = 0.858 |
|  |  |  |  |  |  |  | Relative | 0.0297 | 0.9122 | F = 0.001, df =1,2, P = 0.977 |
| Merulinidae | *Echinopora* | Indo-Pacific | Taiwan outlet | 2007 | 2010 | 4 | Absolute | -0.0559 | 0.0721 | F = 3.000, df =1,2, P = 0.225 |
|  |  |  |  |  |  |  | Relative | -0.1707 | 0.2203 | F = 3.000, df =1,2, P = 0.225 |
| Merulinidae | *Dipsastraea* | Indo-Pacific | Taiwan outlet | 2007 | 2010 | 4 | Absolute | 1.7857 | 0.9498 | F = 17.675, df =1,2, P = 0.052 |
|  |  |  |  |  |  |  | Relative | 4.3747 | 2.3195 | F = 17.786, df =1,2, P = 0.052 |
| Merulinidae | *Favites* | Indo-Pacific | Taiwan outlet | 2007 | 2010 | 4 | Absolute | 1.3440 | 1.0803 | F = 7.738, df =1,2, P = 0.109 |
|  |  |  |  |  |  |  | Relative | 2.4904 | 6.1332 | F = 0.824, df = 1,2, P = 0.460 |
| Euphylliidae | *Galaxea* | Indo-Pacific | Taiwan outlet | 2007 | 2010 | 4 | Absolute | -0.0703 | 1.1373 | F = 0.064, df = 1,2, P = 0.824 |
|  |  |  |  |  |  |  | Relative | -0.9333 | 1.8363 | F = 1.291, df = 1,2, P = 0.374 |
| Merulinidae | *Goniastrea* | Indo-Pacific | Taiwan outlet | 2007 | 2010 | 4 | Absolute | -1.3207 | 2.5091 | F = 1.385, df = 1,2, P = 0.360 |
|  |  |  |  |  |  |  | Relative | -4.3100 | 6.6150 | F = 2.123, df = 1,2, P = 0.283 |
| Poritidae | *Goniopora* | Indo-Pacific | Taiwan outlet | 2007 | 2010 | 4 | Absolute | -0.0795 | 0.1837 | F = 0.937, df = 1,2, P = 0.435 |
|  |  |  |  |  |  |  | Relative | -0.2673 | 0.5421 | F = 1.216, df = 1,2, P = 0.385 |
| Faviidae | *Herpolitha* | Indo-Pacific | Taiwan outlet | 2007 | 2010 | 4 | Absolute | 0.0028 | 0.0036 | F = 3.000, df = 1,2, P = 0.225 |
|  |  |  |  |  |  |  | Relative | 0.0071 | 0.0092 | F = 3.000, df = 1,2, P = 0.225 |
| Merulinidae | *Hydnophora* | Indo-Pacific | Taiwan outlet | 2007 | 2010 | 4 | Absolute | -0.1453 | 0.0906 | F = 12.859, df = 1,2, P = 0.070 |
|  |  |  |  |  |  |  | Relative | -0.4645 | 0.3650 | F = 8.098, df = 1,2, P = 0.105 |
| Incertae sedis | *Leptastrea* | Indo-Pacific | Taiwan outlet | 2007 | 2010 | 4 | Absolute | -0.0340 | 0.1612 | F = 0.222, df = 1,2, P = 0.684 |
|  |  |  |  |  |  |  | Relative | -0.1241 | 0.6089 | F = 0.208, df = 1,2, P = 0.693 |
| Merulinidae | *Leptoria* | Indo-Pacific | Taiwan outlet | 2007 | 2010 | 4 | Absolute | -0.1839 | 0.1922 | F = 4.576, df = 1,2, P = 0.166 |
|  |  |  |  |  |  |  | Relative | -0.5861 | 0.5314 | F = 6.082, df = 1,2, P = 0.133 |
| Lobophylliidae | *Lobophyllia* | Indo-Pacific | Taiwan outlet | 2007 | 2010 | 4 | Absolute | 0.1770 | 0.3127 | F = 1.602, df = 1,2, P = 0.333 |
|  |  |  |  |  |  |  | Relative | 0.4108 | 0.9558 | F = 0.923, df = 1,2, P = 0.438 |
| Merulinidae | *Merulina* | Indo-Pacific | Taiwan outlet | 2007 | 2010 | 4 | Absolute | 0.1450 | 0.2132 | F = 2.315, df = 1,2, P = 0.266 |
|  |  |  |  |  |  |  | Relative | 0.3659 | 0.5517 | F = 2.199, df = 1,2, P = 0.276 |
| Merulinidae | *Phymastrea* | Indo-Pacific | Taiwan outlet | 2007 | 2010 | 4 | Absolute | -0.1261 | 0.1809 | F = 2.430, df = 1,2, P = 0.259 |
|  |  |  |  |  |  |  | Relative | -0.3893 | 0.5489 | F = 2.515, df = 1,2, P = 0.254 |
| Acroporidae | *Montipora* | Indo-Pacific | Taiwan outlet | 2007 | 2010 | 4 | Absolute | -1.1139 | 2.3077 | F = 1.165, df = 1,2, P = 0.393 |
|  |  |  |  |  |  |  | Relative | -4.6441 | 4.2677 | F = 5.921, df = 1,2, P = 0.135 |
| Merulinidae | *Oulophyllia* | Indo-Pacific | Taiwan outlet | 2007 | 2010 | 4 | Absolute | 0.0039 | 0.0234 | F = 0.143, df = 1,2, P = 0.742 |
|  |  |  |  |  |  |  | Relative | 0.0099 | 0.0583 | F = 0.143, df = 1,2, P = 0.742 |
| Lobophylliidae | *Oxypora* | Indo-Pacific | Taiwan outlet | 2007 | 2010 | 4 | Absolute | 0.0038 | 0.0388 | F = 0.049, df = 1,2, P = 0.845 |
|  |  |  |  |  |  |  | Relative | 0.0060 | 0.1022 | F = 0.017, df = 1,2, P = 0.907 |
| Agaricidae | *Pachyseris* | Indo-Pacific | Taiwan outlet | 2007 | 2010 | 4 | Absolute | 0.0002 | 0.0405 | F < 0.001, df = 1,2, P = 0.993 |
|  |  |  |  |  |  |  | Relative | -0.0034 | 0.1013 | F = 0.006, df = 1,2, P = 0.947 |
| Agaricidae | *Pavona* | Indo-Pacific | Taiwan outlet | 2007 | 2010 | 4 | Absolute | 0.0226 | 0.0456 | F = 1.224, df = 1,2, P = 0.384 |
|  |  |  |  |  |  |  | Relative | 0.0531 | 0.1223 | F = 0.945, df = 1,2, P = 0.434 |
| Merulinida | *Platygyra* | Indo-Pacific | Taiwan outlet | 2007 | 2010 | 4 | Absolute | **1.2498** | **0.1591** | **F = 308.525, df = 1,2, P = 0.003** |
|  |  |  |  |  |  |  | Relative | **2.9648** | **1.4216** | **F = 21.748, df = 1,2, P = 0.043** |
| Incertae sedis | *Plesiastrea* | Indo-Pacific | Taiwan outlet | 2007 | 2010 | 4 | Absolute | -0.0056 | 0.0329 | F = 0.143, df = 1,2, P = 0.742 |
|  |  |  |  |  |  |  | Relative | -0.0207 | 0.1227 | F = 0.143, df = 1,2, P = 0.742 |
| Pocilloporidae | *Pocillopora* | Indo-Pacific | Taiwan outlet | 2007 | 2010 | 4 | Absolute | 0.0722 | 0.3765 | F = 0.184, df = 1,2, P = 0.710 |
|  |  |  |  |  |  |  | Relative | -0.0272 | 0.7713 | F = 0.006, df = 1,2, P = 0.944 |
| Poritidae | *Porites* | Indo-Pacific | Taiwan outlet | 2007 | 2010 | 4 | Absolute | 0.5269 | 0.8350 | F = 1.991, df = 1,2, P = 0.294 |
|  |  |  |  |  |  |  | Relative | 0.0050 | 0.0082 | F = 1.894, df = 1,2, P = 0.303 |
| Psammocoridae | *Psammocora* | Indo-Pacific | Taiwan outlet | 2007 | 2010 | 4 | Absolute | 0.0195 | 1.0987 | F = 0.002, df = 1,2, P = 0.972 |
|  |  |  |  |  |  |  | Relative | -0.1758 | 3.0845 | F = 0.016, df = 1,2, P = 0.910 |
| Merulinidae | *Scapophyllia* | Indo-Pacific | Taiwan outlet | 2007 | 2010 | 4 | Absolute | -0.0758 | 0.0858 | F = 3.900, df = 1,2, P = 0.187 |
|  |  |  |  |  |  |  | Relative | -0.2551 | 0.3770 | F = 2.289, df = 1,2, P = 0.270 |
| Pocilloporidae | *Seriatopora* | Indo-Pacific | Taiwan outlet | 2007 | 2010 | 4 | Absolute | 0.0361 | 0.0466 | F = 3.000, df = 1,2, P = 0.225 |
|  |  |  |  |  |  |  | Relative | 0.0926 | 0.1195 | F = 3.000, df = 1,2, P = 0.225 |
| Pocilloporidae | *Stylocoeniella* | Indo-Pacific | Taiwan outlet | 2007 | 2010 | 4 | Absolute | 0.0139 | 0.0179 | F = 3.000, df = 1,2, P = 0.2254 |
|  |  |  |  |  |  |  | Relative | 0.0356 | 0.0460 | F = 3.000, df = 1,2, P = 0.225 |
| Lobophylliidae | *Symphyllia* | Indo-Pacific | Taiwan outlet | 2007 | 2010 | 4 | Absolute | 0.0734 | 0.2791 | F = 0.346, df = 1,2, P = 0.616 |
|  |  |  |  |  |  |  | Relative | 0.1645 | 0.6900 | F = 0.284, df = 1,2, P = 0.647 |
| Dendrophyllidae | *Turbinaria* | Indo-Pacific | Taiwan outlet | 2007 | 2010 | 4 | Absolute | 0.0036 | 0.0641 | F = 0.016, df = 1,2, P = 0.912 |
|  |  |  |  |  |  |  | Relative | 0.0005 | 0.1845 | F < 0.001, df = 1,2, P = 0.999 |
| Acroporidae | *Acropora* | Indo-Pacific | Taiwan Lungken | 2003 | 2010 | 7 | Absolute | 0.0174 | 0.0285 | F = 5.456, df = 1,5, P = 0.067 |
|  |  |  |  |  |  |  | Relative | 0.0304 | 0.8396 | F = 0.046, df = 1,5, P = 0.839 |
| Merulinidae | *Cyphastrea* | Indo-Pacific | Taiwan Lungken | 2003 | 2010 | 7 | Absolute | **0.1214** | **0.0601** | **F = 37.760, df = 1,5, P = 0.002** |
|  |  |  |  |  |  |  | Relative | -0.0096 | 0.9784 | F = 0.003, df = 1,5, P = 0.956 |
| Lobophylliidae | *Echinophyllia* | Indo-Pacific | Taiwan Lungken | 2003 | 2010 | 7 | Absolute | -0.0027 | 0.0116 | F = 2.469, df = 1,5, P = 0.177 |
|  |  |  |  |  |  |  | Relative | -0.2878 | 1.0811 | F = 2.469, df = 1,5, P = 0.177 |
| Merulinidae | *Dipsastraea* | Indo-Pacific | Taiwan Lungken | 2003 | 2010 | 7 | Absolute | **0.0942** | **0.1018** | **F = 95.170, df = 1,5, P < 0.001** |
|  |  |  |  |  |  |  | Relative | -0.5099 | 2.8647 | F = 4.536, df =1,5, P = 0.086 |
| Merulinidae | *Favites* | Indo-Pacific | Taiwan Lungken | 2003 | 2010 | 7 | Absolute | **0.5541** | **0.2520** | **F = 60.905, df = 1,5, P < 0.001** |
|  |  |  |  |  |  |  | Relative | -2.5420 | 6.0540 | F = 0.191, df =1,5, P = 0.681 |
| Merulinidae | *Goniastrea* | Indo-Pacific | Taiwan Lungken | 2003 | 2010 | 7 | Absolute | **1.2549** | **1.1560** | **F = 17.326, df = 1,5, P = 0.009** |
|  |  |  |  |  |  |  | Relative | 0.8444 | 12.6152 | F = 0.044, df = 1,5, P = 0.842 |
| Poritidae | *Goniopora* | Indo-Pacific | Taiwan Lungken | 2003 | 2010 | 7 | Absolute | 0.9836 | 1.2769 | F = 0.247, df = 1,5, P = 0.640 |
|  |  |  |  |  |  |  | Relative | 4.0591 | 10.1926 | F = 0.213, df = 1,5, P = 0.664 |
| Merulinidae | *Hydnopora* | Indo-Pacific | Taiwan Lungken | 2003 | 2010 | 7 | Absolute | **0.1315** | **0.1072** | **F = 36.855, df = 1,5, P = 0.002** |
|  |  |  |  |  |  |  | Relative | -0.2180 | 3.8959 | F = 4.406, df =1,5, P = 0.090 |
| Acroporidae | *Montipora* | Indo-Pacific | Taiwan Lungken | 2003 | 2010 | 7 | Absolute | 0.1875 | 0.4048 | F = 1.161, df = 1,5, P = 0.3304 |
|  |  |  |  |  |  |  | Relative | -2.5762 | 15.1168 | F = 0.855, df = 1,5, P = 0.398 |
| Merulinidae | *Platygyra* | Indo-Pacific | Taiwan Lungken | 2003 | 2010 | 7 | Absolute | **0.0646** | **0.0659** | **F = 58.816, df = 1,5, P < 0.001** |
|  |  |  |  |  |  |  | Relative | **0.2709** | **0.4548** | **F = 15.385, df =1,5, P = 0.011** |
| Pocilloporidae | *Pocillopora* | Indo-Pacific | Taiwan Lungken | 2003 | 2010 | 7 | Absolute | **0.1018** | **0.0731** | **F = 12.630, df =1,5, P = 0.016** |
|  |  |  |  |  |  |  | Relative | -0.5702 | 2.0385 | F = 1.594, df =1,5, P = 0.263 |
| Poritidae | *Porites* | Indo-Pacific | Taiwan Lungken | 2003 | 2010 | 7 | Absolute | **0.1259** | **0.1594** | **F = 18.085, df =1,5, P = 0.008** |
|  |  |  |  |  |  |  | Relative | 0.0298 | 1.9723 | F = 0.136, df =1,5, P = 0.728 |
| Lobophylliidae | *Acanthastrea* | Indo-Pacific | Kenya | 1991 | 2011 | 19 | Absolute | 0.0010 | 0.0080 | F = 0.918, df = 1,17, P = 0.351 |
|  |  |  |  |  |  |  | Relative | 0.0060 | 0.0350 | F = 1.003, df = 1,17, P = 0.331 |
| Acroporidae | *Acropora* | Indo-Pacific | Kenya | 1991 | 2011 | 19 | Absolute | **-0.1350** | **0.0320** | **F = 7.557, df = 1,17, P = 0.014** |
|  |  |  |  |  |  |  | Relative | **-0.5510** | **1.2020** | **F = 12.102, df = 1,17, P = 0.003** |
| Poritidae | *Alveopora* | Indo-Pacific | Kenya | 1991 | 2011 | 19 | Absolute | **0.0030** | **0.0080** | **F = 6.386, df = 1,17, P= 0.022** |
|  |  |  |  |  |  |  | Relative | 0.0110 | 0.0430 | F = 2.267, df = 1,17, P = 0.151 |
| Acroporidae | *Astreopora* | Indo-Pacific | Kenya | 1991 | 2011 | 19 | Absolute | **-0.0130** | **0.0310** | **F = 9.045, df = 1,17, P = 0.008** |
|  |  |  |  |  |  |  | Relative | **-0.0550** | **0.1640** | **F = 4.825, df = 1,17, P = 0.042** |
| Coscinaraeidae | *Coscinaraea* | Indo-Pacific | Kenya | 1991 | 2011 | 19 | Absolute | 0.0010 | 0.0030 | F = 1.519, df = 1,17, P = 0.235 |
|  |  |  |  |  |  |  | Relative | 0.0030 | 0.0200 | F = 0.651, df = 1,17, P = 0.431 |
| Merulinidae | *Cyphastrea* | Indo-Pacific | Kenya | 1991 | 2011 | 19 | Absolute | -0.0003 | 0.0130 | F = 0.020, df = 1,17, P = 0.889 |
|  |  |  |  |  |  |  | Relative | -0.0100 | 0.0950 | F = 0.366, df = 1,17, P = 0.533 |
| Lobophyllidae | *Echinophyllia* | Indo-Pacific | Kenya | 1991 | 2011 | 19 | Absolute | 0.0000 | 0.0020 | F = 0.022, df = 1,17, P = 0.883 |
|  |  |  |  |  |  |  | Relative | 0.0002 | 0.0150 | F = 0.004, df = 1,17, P =0.949 |
| Merulinidae | *Echinopora* | Indo-Pacific | Kenya | 1991 | 2011 | 19 | Absolute | **0.0580** | **0.1660** | **F = 16.313, df = 1,17, P = 0.001** |
|  |  |  |  |  |  |  | Relative | 0.1380 | 0.5680 | F = 2.268, df = 1,17, P = 0.150 |
| Merulinidae | *Dipsastraea* | Indo-Pacific | Kenya | 1991 | 2011 | 19 | Absolute | 0.0350 | 0.0330 | F = 0.613, df =1,17, P = 0.445 |
|  |  |  |  |  |  |  | Relative | -0.0420 | 0.1900 | F = 1.847, df = 1,17, P = 0.192 |
| Merulinidae | *Favites* | Indo-Pacific | Kenya | 1991 | 2011 | 19 | Absolute | 0.0050 | 0.0340 | F = 0.656, df = 1,17, P = 0.429 |
|  |  |  |  |  |  |  | Relative | 0.0030 | 0.1610 | F = 0.009, df = 1,17, P = 0.926 |
| Fungidae | *Fungia* | Indo-Pacific | Kenya | 1991 | 2011 | 19 | Absolute | **0.0050** | **0.0100** | **F = 13.408, df = 1,17, P = 0.002** |
|  |  |  |  |  |  |  | Relative | **0.0170** | **0.0470** | **F = 6.171, df = 1,17, P = 0.024** |
| Euphylliidae | *Galaxea* | Indo-Pacific | Kenya | 1991 | 2011 | 19 | Absolute | 0.0080 | 0.0530 | F = 0.789, df = 1,17, P = 0.387 |
|  |  |  |  |  |  |  | Relative | 0.0110 | 0.2370 | F = 0.058, df = 1,17, P = 0.812 |
| Agaricidae | *Gardineroseris* | Indo-Pacific | Kenya | 1991 | 2011 | 19 | Absolute | 0.0003 | 0.0010 | F = 2.820, df = 1,17, P = 0.111 |
|  |  |  |  |  |  |  | Relative | 0.0010 | 0.0050 | F = 2.711, df = 1,17, P = 0.118 |
| Merulinidae | *Goniastrea* | Indo-Pacific | Kenya | 1991 | 2011 | 19 | Absolute | **0.0080** | **0.0230** | **F = 5.045, df = 1,17, P = 0.038** |
|  |  |  |  |  |  |  | Relative | 0.0200 | 0.0910 | F = 1.714, df = 1,17, P = 0.208 |
| Poritidae | *Goniopora* | Indo-Pacific | Kenya | 1991 | 2011 | 19 | Absolute | -0.0040 | 0.0200 | F = 1.561, df = 1,17, P = 0.228 |
|  |  |  |  |  |  |  | Relative | -0.0210 | 0.0820 | F = 2.623, df = 1,17, P = 0.124 |
| Faviidae | *Herpolitha* | Indo-Pacific | Kenya | 1991 | 2011 | 19 | Absolute | 0.0003 | 0.0020 | F = 0.516, df = 1,17, P = 0.482 |
|  |  |  |  |  |  |  | Relative | 0.0010 | 0.0090 | F = 0.515, df = 1,17, P = 0.483 |
| Merulinidae | *Hydnophora* | Indo-Pacific | Kenya | 1991 | 2011 | 19 | Absolute | -0.0050 | 0.0340 | F = 0.901, df = 1,17, P = 0.356 |
|  |  |  |  |  |  |  | Relative | -0.0340 | 0.1610 | F = 1.624, df = 1,17, P = 0.220 |
| Incertae sedis | *Leptastrea* | Indo-Pacific | Kenya | 1991 | 2011 | 19 | Absolute | -0.0030 | 0.0280 | F = 0.480, df = 1,17, P = 0.498 |
|  |  |  |  |  |  |  | Relative | -0.0100 | 0.0970 | F = 0.375, df = 1,17, P = 0.158 |
| Merulinidae | *Leptoria* | Indo-Pacific | Kenya | 1991 | 2011 | 19 | Absolute | 0.0060 | 0.0260 | F = 2.229, df = 1,17, P = 0.154 |
|  |  |  |  |  |  |  | Relative | 0.0230 | 0.1130 | F = 1.536, df = 1,17, P = 0.232 |
| Lobophylliidae | *Lobophyllia* | Indo-Pacific | Kenya | 1991 | 2011 | 19 | Absolute | 0.0010 | 0.0050 | F = 3.516, df = 1,17, P = 0.077 |
|  |  |  |  |  |  |  | Relative | 0.0005 | 0.0080 | F = 0.374, df = 1,17, P = 0.713 |
| Merulinidae | *Phymastrea* | Indo-Pacific | Kenya | 1991 | 2011 | 19 | Absolute | 0.0010 | 0.0050 | F = 2.070, df = 1,17, P = 0.168 |
|  |  |  |  |  |  |  | Relative | 0.0050 | 0.0200 | F = 1.896, df = 1,17, P = 0.186 |
| Acroporidae | *Montipora* | Indo-Pacific | Kenya | 1991 | 2011 | 19 | Absolute | **-0.2120** | **0.3700** | **F = 31.659, df = 1,17, P < 0.001** |
|  |  |  |  |  |  |  | Relative | **-0.8690** | **1.3570** | **F = 74.480, df = 1,17, P < 0.001** |
| Lobophylliidae | *Oxypora* | Indo-Pacific | Kenya | 1991 | 2011 | 19 | Absolute | 0.0001 | 0.0004 | F = 0.749, df = 1,17, P = 0.400 |
|  |  |  |  |  |  |  | Relative | 0.0003 | 0.0020 | F = 0.749, df = 1,17, P = 0.400 |
| Agaricidae | *Pavona* | Indo-Pacific | Kenya | 1991 | 2011 | 19 | Absolute | **0.1200** | **0.1910** | **F = 62.795, df = 1,17, P <0.001** |
|  |  |  |  |  |  |  | Relative | **0.3310** | **0.8540** | **F = 7.216, df = 1,17, P = 0.016** |
| Merulinidae | *Platygyra* | Indo-Pacific | Kenya | 1991 | 2011 | 19 | Absolute | 0.0050 | 0.0280 | F = 1.199, df = 1,17, P = 0.289 |
|  |  |  |  |  |  |  | Relative | -0.0100 | 0.1450 | F = 0.154, df = 1,17, P = 0.619 |
| Pocilloporidae | *Pleisiastrea* | Indo-Pacific | Kenya | 1991 | 2011 | 19 | Absolute | -0.0003 | 0.0020 | F = 0.789, df = 1,17, P = 0.387 |
|  |  |  |  |  |  |  | Relative | -0.0010 | 0.0080 | F = 0.605, df = 1,17, P = 0.447 |
| Pocilloporidae | *Pocillopora* | Indo-Pacific | Kenya | 1991 | 2011 | 19 | Absolute | **0.0490** | **0.1120** | **F = 10.645, df = 1,17, P = 0.005** |
|  |  |  |  |  |  |  | Relative | **0.1690** | **0.5110** | **F = 4.712, df = 1,17, P = 0.044** |
| Poritidae | *Porites* | Indo-Pacific | Kenya | 1991 | 2011 | 19 | Absolute | -0.0100 | 0.0870 | F = 0.015, df = 1,17, P = 0.904 |
|  |  |  |  |  |  |  | Relative | 0.0570 | 0.2830 | F = 0.040, df = 1,17, P = 0.843 |
| Psammocoridae | *Psammocora* | Indo-Pacific | Kenya | 1991 | 2011 | 19 | Absolute | 0.0030 | 0.0120 | F = 2.619, df = 1,17, P = 0.124 |
|  |  |  |  |  |  |  | Relative | -0.0002 | 0.0330 | F = 0.002, df = 1,17, P = 0.967 |
| Pocilloporidae | *Seriatopora* | Indo-Pacific | Kenya | 1991 | 2011 | 19 | Absolute | 0.0120 | 0.0630 | F = 1.297, df = 1,17, P = 0.271 |
|  |  |  |  |  |  |  | Relative | 0.0520 | 0.2760 | F = 1.283, df = 1,17, P = 0.273 |
| Pocilloporidae | *Stylophora* | Indo-Pacific | Kenya | 1991 | 2011 | 19 | Absolute | -0.0340 | 0.0610 | F = 1.626, df = 1,17, P = 0.219 |
|  |  |  |  |  |  |  | Relative | **-0.2750** | **0.6180** | **F = 10.928, df = 1,17, P = 0.004** |
| Poritidae | *Synarea* | Indo-Pacific | Kenya | 1991 | 2011 | 19 | Absolute | **0.0540** | **0.0940** | **F = 31.683, df = 1,17, P < 0.001** |
|  |  |  |  |  |  |  | Relative | **0.1870** | **0.3940** | **F = 13.575, df = 1,17, P = 0.002** |
| Dendrophyllidae | *Tubastrea* | Indo-Pacific | Kenya | 1991 | 2011 | 19 | Absolute | -0.0010 | 0.0040 | F = 1.638, df = 1,17, P = 0.218 |
|  |  |  |  |  |  |  | Relative | -0.0030 | 0.0130 | F = 1.686, df = 1,17, P = 0.211 |
| Dendrophyllidae | *Turbinaria* | Indo-Pacific | Kenya | 1991 | 2011 | 19 | Absolute | 0.0010 | 0.0080 | F = 1.140, df = 1,17, P = 0.301 |
|  |  |  |  |  |  |  | Relative | 0.0060 | **0.0320** | F = 1.498, df = 1,17, P = 0.238 |
| Acroporidae | *Acropora* | Caribbean | Belize | 1996 | 2010 | 10 | Absolute | -0.0100 | 0.0678 | F = 0.661, df = 1,9, P = 0.525 |
|  |  |  |  |  |  |  | Relative | -0.0400 | 0.3815 | F = 0.218, df = 1,9, P = 0.652 |
| Agaricidae | *Agaricia* | Caribbean | Belize | 1996 | 2010 | 10 | Absolute | 0.0150 | 0.3729 | F = 0.173, df = 1,9, P = 0.867 |
|  |  |  |  |  |  |  | Relative | 0.3340 | 1.4379 | F = 1.944, df = 1,9, P = 0.303 |
| Mussidae | *Colpophyllia* | Caribbean | Belize | 1996 | 2010 | 10 | Absolute | -0.0020 | 0.0180 | F = 0.163, df = 1,9, P = 0.696 |
|  |  |  |  |  |  |  | Relative | -0.0100 | 0.1162 | F = 0.142, df = 1,9, P = 0.716 |
| Mussidae | *Diploria* | Caribbean | Belize | 1996 | 2010 | 10 | Absolute | -0.0100 | 0.0588 | F = 0.593, df = 1,9, P = 0.461 |
|  |  |  |  |  |  |  | Relative | 0.0080 | 0.4532 | F = 0. 005, df = 1,9, P = 0.943 |
| Meandrinidae | *Eusmilia* | Caribbean | Belize | 1996 | 2010 | 10 | Absolute | 0.0030 | 0.0120 | F = 1.316, df = 1,9, P = 0.281 |
|  |  |  |  |  |  |  | Relative | 0.0140 | 0.0722 | F = 0.769, df = 1,9, P = 0.743 |
| Mussidae | *Favia* | Caribbean | Belize | 1996 | 2010 | 10 | Absolute | -0.0060 | 0.0170 | F = 3.074, df = 1,9, P = 0.113 |
|  |  |  |  |  |  |  | Relative | -0.0340 | 0.1139 | F = 2.143, df = 1,9, P = 0.177 |
| Mussidae | *Manicina* | Caribbean | Belize | 1996 | 2010 | 10 | Absolute | -0.0030 | 0.0101 | F = 1.481, df = 1,9, P = 0.255 |
|  |  |  |  |  |  |  | Relative | -0.0090 | 0.0466 | F = 0.877, df = 1,9, P = 0.374 |
| Montastreidae | *Montastraea* | Caribbean | Belize | 1996 | 2010 | 10 | Absolute | 0.0010 | 0.0330 | F = 0.013, df = 1,9, P = 0.911 |
|  |  |  |  |  |  |  | Relative | 0.0220 | 0.2452 | F = 0.167, df = 1,9, P = 0.692 |
| Merulinidae | *Orbicella* | Caribbean | Belize | 1996 | 2010 | 10 | Absolute | -0.2370 | 0.5419 | **F = 6.402, df = 1,9, P = 0.032** |
|  |  |  |  |  |  |  | Relative | -0.9250 | 2.4330 | F = 4.111, df = 1,9, P = 0.073 |
| Mussidae | *Mussa* | Caribbean | Belize | 1996 | 2010 | 10 | Absolute | 0.0020 | 0.0071 | F = 3.096, df = 1,9, P = 0.112 |
|  |  |  |  |  |  |  | Relative | 0.0120 | 0.0384 | F = 2.573, df = 1,9, P = 0.143 |
| Poritidae | *Porites* | Caribbean | Belize | 1996 | 2010 | 10 | Absolute | 0.0269 | 0.0695 | F = 0.150, df = 1,9, P = 0.707 |
|  |  |  |  |  |  |  | Relative | 0.4320 | 0.286 | F = 2.278, df = 1,9, P = 0.165 |
| Mussidae | *Scolymia* | Caribbean | Belize | 1996 | 2010 | 10 | Absolute | -0.0010 | 0.0040 | F = 0.373, df = 1,9, P = 0.557 |
|  |  |  |  |  |  |  | Relative | -0.0040 | 0.0264 | F = 0.376, df = 1,9, P = 0.555 |
| Siderastreidae | *Siderastrea* | Caribbean | Belize | 1996 | 2010 | 10 | Absolute | 0.0140 | 0.1901 | F = 0.101, df = 1,9, P = 0.758 |
|  |  |  |  |  |  |  | Relative | 0.1560 | 1.2481 | F = 0.314, df = 1,9, P = 0.589 |
| Merulinidae | *Orbicella* | Caribbean | St. Croix ≤ 10 m | 2001 | 2012 | 12 | Absolute | -0.4920 | 0.1076 | **F = 20.919, df = 1, 346 P < 0.001** |
|  |  |  |  |  |  |  | Relative | -0.0047 | 0.0048 | F = 0.9914, df = 1,346, P = 0.320 |
| Montastraeidae | *Montastraea* | Caribbean | St. Croix ≤ 10 m | 2001 | 2012 | 12 | Absolute | -0.0921 | 0.0263 | **F = 12.271, df = 1, 346, P < 0.001** |
|  |  |  |  |  |  |  | Relative | -0.0057 | 0.0026 | **F = 4.790, df = 1, 346, P = 0.029** |
| Agaricidae | *Agaricia* | Caribbean | St. Croix ≤ 10 m | 2001 | 2012 | 12 | Absolute | -0.0836 | 0.0107 | **F = 60.777, df = 1, 346, P < 0.001** |
|  |  |  |  |  |  |  | Relative | -0.0050 | 0.0007 | **F = 46.114, df = 1, 346, P < 0.001** |
| Meandrinidae | *Dichocoenia* | Caribbean | St. Croix ≤ 10 m | 2001 | 2012 | 12 | Absolute | 0.0005 | 0.0012 | F = 0.205, df = 1, 346, P = 0.651 |
|  |  |  |  |  |  |  | Relative | 0.0001 | 0.0002 | F = 0.339, df = 1, 346, P = 0.561 |
| Mussidae | *Diploria* | Caribbean | St. Croix ≤ 10 m | 2001 | 2012 | 12 | Absolute | -0.0157 | 0.0217 | F = 0.523, df = 1, 346, P = 0.470 |
|  |  |  |  |  |  |  | Relative | 0.0027 | 0.0020 | F = 1.750, df = 1, 346, P = 0.187 |
| Meandrinidae | *Meandrina* | Caribbean | St. Croix ≤ 10 m | 2001 | 2012 | 12 | Absolute | -0.0033 | 0.0073 | F = 0.197, df = 1, 346, P = 0.657 |
|  |  |  |  |  |  |  | Relative | 0.0004 | 0.0010 | F = 0.207, df = 1, 346, P = 0.650 |
| Poritidae | *Porites* | Caribbean | St. Croix ≤ 10 m | 2001 | 2012 | 12 | Absolute | -0.0060 | 0.0319 | F = 0.036, df = 1, 346, P = 0.850 |
|  |  |  |  |  |  |  | Relative | 0.0120 | 0.0028 | **F = 17.399, df = 1, 346, P < 0.001** |
| Astrocoeniidae | *Stephanocoenia* | Caribbean | St. Croix ≤ 10 m | 2001 | 2012 | 12 | Absolute | 0.0034 | 0.0030 | F = 1.242, df = 1, 346, P = 0.266 |
|  |  |  |  |  |  |  | Relative | 0.0003 | 0.0003 | F = 0.753, df = 1, 346, P = 0.386 |
| Siderastreidae | *Siderastrea* | Caribbean | St. Croix ≤ 10 m | 2001 | 2012 | 12 | Absolute | -0.0412 | 0.0199 | **F = 4.295, df = 1, 346, P = 0.039** |
|  |  |  |  |  |  |  | Relative | -0.0005 | 0.0019 | F = 0.080, df = 1, 346, P = 0.778 |
| Merulinidae | *Orbicella* | Caribbean | St. Croix 11-25 m | 2003 | 2012 | 12 | Absolute | -1.3035 | 0.2018 | **F = 41.742, df = 1, 172, P < 0.001** |
|  |  |  |  |  |  |  | Relative | -0.0171 | 0.0081 | **F = 4.480, df = 1, 172, P = 0.036** |
| Montastraeidae | *Montastraea* | Caribbean | St. Croix 11-25 m | 2003 | 2012 | 10 | Absolute | -0.0959 | 0.0388 | **F = 6.100, df = 1, 172, P = 0.015** |
|  |  |  |  |  |  |  | Relative | -0.0089 | 0.0058 | F = 2.378, df = 1, 172, P = 0.125 |
| Agaricidae | *Agaricia* | Caribbean | St. Croix 11-25 m | 2003 | 2012 | 10 | Absolute | -0.0115 | 0.0128 | F = 0.797, df = 1, 172, P = 0.373 |
|  |  |  |  |  |  |  | Relative | 0.0021 | 0.0019 | F = 1.268, df = 1, 172, P = 0.262 |
| Meandrinidae | *Dichocoenia* | Caribbean | St. Croix 11-25 m | 2003 | 2012 | 10 | Absolute | -0.0063 | 0.0035 | F = 3.288, df = 1, 172, P = 0.072 |
|  |  |  |  |  |  |  | Relative | -0.0010 | 0.0007 | F = 2.189, df = 1, 172, P = 0.141 |
| Mussidae | *Diploria* | Caribbean | St. Croix 11-25 m | 2003 | 2012 | 10 | Absolute | -0.0245 | 0.0188 | F = 1.736, df = 1, 172, P = 0.189 |
|  |  |  |  |  |  |  | Relative | 0.0010 | 0.0027 | F = 0.123, df = 1, 172, P = 0.720 |
| Meandrinidae | *Meandrina* | Caribbean | St. Croix 11-25 m | 2003 | 2012 | 10 | Absolute | 0.0305 | 0.0108 | **F = 7.956, df = 1, 172, P = 0.005** |
|  |  |  |  |  |  |  | Relative | 0.0076 | 0.0023 | **F = 10.794, df = 1, 172, P = 0.001** |
| Poritidae | *Porites* | Caribbean | St. Croix 11-25 m | 2003 | 2012 | 10 | Absolute | -0.1230 | 0.0371 | **F = 10.977, df = 1, 172, P = 0.001** |
|  |  |  |  |  |  |  | Relative | 0.0069 | 0.0045 | F = 2.302, df = 1, 172, P = 0.131 |
| Astrocoeniidae | *Stephanocoenia* | Caribbean | St. Croix 11-25 m | 2003 | 2012 | 10 | Absolute | -0.0009 | 0.0053 | F = 2.689, df = 1, 172, P = 0.103 |
|  |  |  |  |  |  |  | Relative | -0.0009 | 0.0006 | F = 2.020, df = 1, 172, P = 0.157 |
| Siderastreidae | *Siderastrea* | Caribbean | St. Croix 11-25 m | 2003 | 2012 | 10 | Absolute | -0.0240 | 0.0201 | F = 1.424, df = 1, 172, P = 0.234 |
|  |  |  |  |  |  |  | Relative | 0.0052 | 0.0032 | F = 2.582, df = 1, 172, P = 0.110 |
| Merulinidae | *Orbicella* | Caribbean | N. USVI ≤ 10 m | 2001 | 2012 | 12 | Absolute | -0.2743 | 0.1428 | F = 3.691, df = 1, 226, P = 0.056 |
|  |  |  |  |  |  |  | Relative | 0.0001 | 0.0064 | F < 0.001, df = 1, 226, P = 0.994 |
| Montastraeidae | *Montastraea* | Caribbean | N. USVI ≤ 10 m | 2001 | 2012 | 12 | Absolute | -0.2078 | 0.0320 | **F = 29.882, df = 1, 226, P < 0.001** |
|  |  |  |  |  |  |  | Relative | -0.0112 | 0.0035 | **F = 10.401, df = 1, 226, P = 0.001** |
| Agaricidae | *Agaricia* | Caribbean | N. USVI ≤ 10 m | 2003 | 2012 | 10 | Absolute | -0.0511 | 0.0129 | **F = 15.731, df = 1, 226, P < 0.001** |
|  |  |  |  |  |  |  | Relative | -0.0042 | 0.0017 | **F = 6.553, df = 1, 226, P = 0.011** |
| Meandrinidae | *Dichocoenia* | Caribbean | N. USVI ≤ 10 m | 2003 | 2012 | 10 | Absolute | 0.0020 | 0.0021 | F = 0.913, df = 1, 226, P = 0.340 |
|  |  |  |  |  |  |  | Relative | 0.0004 | 0.0012 | F = 0.112, df = 1, 226, P = 0.739 |
| Mussidae | *Diploria* | Caribbean | N. USVI ≤ 10 m | 2003 | 2012 | 10 | Absolute | -0.0658 | 0.0112 | **F = 6.548, df = 1, 226, P = 0.011** |
|  |  |  |  |  |  |  | Relative | -0.0031 | 0.0021 | F = 2.250, df = 1, 226, P = 0.135 |
| Meandrinidae | *Meandrina* | Caribbean | N. USVI ≤ 10 m | 2003 | 2012 | 10 | Absolute | -0.0019 | 0.0046 | F = 0.160, df = 1, 226, P = 0.689 |
|  |  |  |  |  |  |  | Relative | -0.0001 | 0.0004 | F = 0.062, df = 1, 226, P = 0.804 |
| Poritidae | *Porites* | Caribbean | N. USVI ≤ 10 m | 2003 | 2012 | 10 | Absolute | 0.0179 | 0.0379 | F = 0.224, df = 1, 226, P = 0.637 |
|  |  |  |  |  |  |  | Relative | 0.0126 | 0.0038 | **F = 10.799, df = 1, 226, P = 0.001** |
| Astrocoeniidae | *Stephanocoenia* | Caribbean | N. USVI ≤ 10 m | 2003 | 2012 | 10 | Absolute | 0.0019 | 0.0058 | F = 0.107, df = 1, 226, P = 0.744 |
|  |  |  |  |  |  |  | Relative | 0.0017 | 0.0011 | F = 2.150, df = 1, 226, P = 0.144 |
| Siderastreidae | *Siderastrea* | Caribbean | N. USVI ≤ 10 m | 2003 | 2012 | 10 | Absolute | 0.1262 | 0.0412 | **F = 9.402, df = 1, 226, P = 0.002** |
|  |  |  |  |  |  |  | Relative | 0.0126 | 0.0044 | **F = 8.146, df = 1, 226, P = 0.005** |
| Merulinidae | *Orbicella* | Caribbean | N. USVI 11-25 m | 2003 | 2012 | 10 | Absolute | -0.7946 | 0.1610 | **F = 24.121, df = 1, 298, P < 0.001** |
|  |  |  |  |  |  |  | Relative | -0.0001 | 0.0050 | F = 0.0387, df = 1, 298, P = 0.844 |
| Montastraeidae | *Montastraea* | Caribbean | N. USVI 11-25 m | 2003 | 2012 | 10 | Absolute | -0.0853 | 0.0340 | **F = 6.179, df = 1, 298, P = 0.013** |
|  |  |  |  |  |  |  | Relative | -0.0038 | 0.0020 | F = 3.852, df = 1, 298, P = 0.051 |
| Agaricidae | *Agaricia* | Caribbean | N. USVI 11-25 m | 2003 | 2012 | 10 | Absolute | -0.0452 | 0.0190 | **F = 5.776, df = 1, 298, P = 0.017** |
|  |  |  |  |  |  |  | Relative | -0.0012 | 0.0010 | F = 1.165, df = 1, 298, P = 0.281 |
| Meandrinidae | *Dichocoenia* | Caribbean | N. USVI 11-25 m | 2003 | 2012 | 10 | Absolute | 0.0007 | 0.0001 | F = 0.861, df = 1, 298, P = 0.354 |
|  |  |  |  |  |  |  | Relative | 0.0001 | 0.0001 | F = 0.432, df = 1, 298, P = 0.512 |
| Mussidae | *Diploria* | Caribbean | N. USVI 11-25 m | 2003 | 2012 | 10 | Absolute | -0.0184 | 0.0100 | F = 2.090, df = 1, 298, P = 0.149 |
|  |  |  |  |  |  |  | Relative | -0.0007 | 0.0006 | F = 1.158, df = 1, 298, P = 0.283 |
| Meandrinidae | *Meandrina* | Caribbean | N. USVI 11-25 m | 2003 | 2012 | 10 | Absolute | 0.0089 | 0.0089 | F = 1.007, df = 1, 298, P = 0.317 |
|  |  |  |  |  |  |  | Relative | 0.0004 | 0.0005 | F = 0.660, df = 1, 298, P = 0.417 |
| Poritidae | *Porites* | Caribbean | N. USVI 11-25 m | 2003 | 2012 | 10 | Absolute | -0.0382 | 0.0710 | F = 0.292, df = 1, 298, P = 0.590 |
|  |  |  |  |  |  |  | Relative | -0.0050 | 0.0034 | F = 2.1690, df = 1, 298, P = 0.142 |
| Astrocoeniidae | *Stephanocoenia* | Caribbean | N. USVI 11-25 m | 2003 | 2012 | 10 | Absolute | 0.0086 | 0.0050 | **F = 2.732, df = 1, 298, P = 0.099** |
|  |  |  |  |  |  |  | Relative | 0.0006 | 0.0004 | F = 2.430, df = 1, 298, P = 0.120 |
| Siderastreidae | *Siderastrea* | Caribbean | N. USVI 11-25 m | 2003 | 2012 | 10 | Absolute | 0.0839 | 0.0290 | **F = 8.390, df = 1, 298, P = 0.004** |
|  |  |  |  |  |  |  | Relative | 0.0052 | 0.0052 | **F = 9.549, df = 1, 298, P = 0.002** |
| Merulinidae | *Orbicella* | Caribbean | N. USVI >25 m | 2003 | 2012 | 10 | Absolute | -0.4692 | 0.2800 | F = 2.809, df = 1, 179, P = 0.096 |
|  |  |  |  |  |  |  | Relative | 0.0013 | 0.0031 | F = 0.172, df = 1, 179, P = 0.679 |
| Montastraeidae | *Montastraea* | Caribbean | N. USVI >25 m | 2003 | 2012 | 10 | Absolute | -0.0086 | 0.0086 | F = 0.163, df = 1, 179, P = 0.687 |
|  |  |  |  |  |  |  | Relative | 0.0001 | 0.0007 | F = 0.005, df = 1, 179, P = 0.945 |
| Agaricidae | *Agaricia* | Caribbean | N. USVI >25 m | 2003 | 2012 | 10 | Absolute | 0.1415 | 0.0537 | **F = 6.936, df = 1, 179, P = 0.009** |
|  |  |  |  |  |  |  | Relative | 0.0046 | 0.0020 | **F = 5.173, df = 1, 179, P = 0.024** |
| Meandrinidae | *Dichocoenia* | Caribbean | N. USVI >25 m | 2003 | 2012 | 10 | Absolute | 0.0002 | 0.0014 | F = 0.014, df = 1, 179, P = 0.905 |
|  |  |  |  |  |  |  | Relative | -0.0001 | 0.0001 | F = 0.111, df = 1, 179, P = 0.739 |
| Mussidae | *Diploria* | Caribbean | N. USVI >25 m | 2003 | 2012 | 10 | Absolute | -0.0445 | 0.0240 | F = 3.448, df = 1, 179, P = 0.065 |
|  |  |  |  |  |  |  | Relative | -0.0014 | 0.0009 | F = 1.998, df = 1, 179, P = 0.159 |
| Meandrinidae | *Meandrina* | Caribbean | N. USVI >25 m | 2003 | 2012 | 10 | Absolute | 0.0026 | 0.0026 | F = 1.142, df = 1, 179, P = 0.227 |
|  |  |  |  |  |  |  | Relative | 0.0001 | 0.0001 | F = 1.419, df = 1, 179, P = 0.235 |
| Poritidae | *Porites* | Caribbean | N. USVI >25 m | 2003 | 2012 | 10 | Absolute | -0.0334 | 0.0350 | F = 0.915, df = 1, 179, P = 0.340 |
|  |  |  |  |  |  |  | Relative | 0.0001 | 0.0011 | F = 0.002, df = 1, 179, P = 0.968 |
| Astrocoeniidae | *Stephanocoenia* | Caribbean | N. USVI >25 m | 2003 | 2012 | 10 | Absolute | 0.0107 | 0.0037 | **F = 8.562, df = 1, 179, P = 0.004** |
|  |  |  |  |  |  |  | Relative | 0.0004 | 0.0001 | **F = 6.523, df = 1, 179, P = 0.012** |
| Siderastreidae | *Siderastrea* | Caribbean | N. USVI >25 m | 2003 | 2012 | 10 | Absolute | -0.0200 | 0.0244 | F = 0.672, df = 1, 179, P = 0.413 |
|  |  |  |  |  |  |  | Relative | -0.0003 | 0.0007 | F = 0.203, df = 1, 179, P = 0.653 |
| Acroporidae | *Acropora* | Indo-Pacific | GBR Inshore | 1995 | 2013 | 15 | Absolute | -0.0960 | 0.0480 | F = 4.000. df = 1,13, P = 0.067 |
|  |  |  |  |  |  |  | Relative | -0.0026 | 0.0009 | **F = 7.900, df = 1, 13, P = 0.015** |
| Merulinidae | *Echinopora* | Indo-Pacific | GBR Inshore | 1995 | 2013 | 15 | Absolute | -0.0232 | 0.0100 | **F = 5.390, df = 1,13, P = 0.037** |
|  |  |  |  |  |  |  | Relative | -0.0006 | 0.0003 | F = 3.580, df = 1, 13, P = 0.081 |
| Merulinidae | *Goniastrea* | Indo-Pacific | GBR Inshore | 1995 | 2013 | 15 | Absolute | -0.0027 | 0.0027 | F = 0.940, df = 1,13, P = 0.349 |
|  |  |  |  |  |  |  | Relative | -0.0001 | 0.0001 | F = 0.630, df = 1, 13, P = 0.441 |
| Acroporidae | *Isopora* | Indo-Pacific | GBR Inshore | 1995 | 2013 | 15 | Absolute | -0.0076 | 0.0061 | F = 1.580, df = 1,13, P = 0.232 |
|  |  |  |  |  |  |  | Relative | -0.0002 | 0.0002 | F = 1.380, df = 1, 13, P = 0.261 |
| Incertae sedis | *Leptastrea* | Indo-Pacific | GBR Inshore | 1995 | 2013 | 15 | Absolute | 0.0016 | 0.0010 | F = 2.290, df = 1,13, P = 0.154 |
|  |  |  |  |  |  |  | Relative | 0.0001 | 0.0000 | F = 2.910, df = 1, 13, P = 0.112 |
| Merulinidae | *Phymastrea* | Indo-Pacific | GBR Inshore | 1995 | 2013 | 15 | Absolute | 0.0017 | 0.0007 | **F = 5.450, df = 1,13, P = 0.036** |
|  |  |  |  |  |  |  | Relative | 0.0001 | 0.0000 | **F = 5.300, df = 1, 13, P = 0.039** |
| Acroporidae | *Montipora* | Indo-Pacific | GBR Inshore | 1995 | 2013 | 15 | Absolute | -0.0126 | 0.0196 | F = 0.410, df = 1,13, P = 0.533 |
|  |  |  |  |  |  |  | Relative | 0.0000 | 0.0004 | F = 0.010, df = 1, 13, P = 0.927 |
| Agaricidae | *Pavona* | Indo-Pacific | GBR Inshore | 1995 | 2013 | 15 | Absolute | 0.0120 | 0.0037 | **F = 10.530, df = 1,13, P = 0.006** |
|  |  |  |  |  |  |  | Relative | 0.0004 | 0.0001 | **F = 17.940, df = 1, 13, P = 0.001** |
| Pocilloporidae | *Pocillopora* | Indo-Pacific | GBR Inshore | 1995 | 2013 | 15 | Absolute | 0.0160 | 0.0066 | **F = 5.800, df = 1,13, P = 0.032** |
|  |  |  |  |  |  |  | Relative | 0.0006 | 0.0002 | **F = 13.050, df = 1, 13, P = 0.003** |
| Poritidae | *Porites* | Indo-Pacific | GBR Inshore | 1995 | 2013 | 15 | Absolute | 0.128 | 0.020 | **F = 39.860, df = 1, 13, P = 0.010** |
|  |  |  |  |  |  |  | Relative | 0.005 | 0.001 | **F = 36.839, df = 1, 13, P = 0.000** |
| Pocilloporidae | *Seriatopora* | Indo-Pacific | GBR Inshore | 1995 | 2013 | 15 | Absolute | -0.0221 | 0.0094 | **f = 5.610, df = 1,13, P = 0.034** |
|  |  |  |  |  |  |  | Relative | -0.0006 | 0.0002 | **F = 7.210, df = 1, 13, P = 0.019** |
| Pocilloporidae | *Stylophora* | Indo-Pacific | GBR Inshore | 1995 | 2013 | 15 | Absolute | -0.0077 | 0.0043 | F = 3.220, df = 1,13, P = 0.096 |
|  |  |  |  |  |  |  | Relative | -0.0002 | 0.0001 | F = 4.180, df = 1, 13, P = 0.062 |
| Acroporidae | *Acropora* | Indo-Pacific | GBR Mid-shelf | 1995 | 2013 | 15 | Absolute | -0.2123 | 0.0661 | **F = 10.320, df = 1,13, P = 0.007** |
|  |  |  |  |  |  |  | Relative | -0.0057 | 0.0015 | **F = 15.290, df = 1, 13, P = 0.002** |
| Merulinidae | *Echinopora* | Indo-Pacific | GBR Mid-shelf | 1995 | 2013 | 15 | Absolute | 0.0289 | 0.0063 | **F = 21.090, df = 1, 13, P = 0.001** |
|  |  |  |  |  |  |  | Relative | 0.0014 | 0.0002 | **F = 35.060, df = 1, 13, P < 0.001** |
| Merulinidae | *Goniastrea* | Indo-Pacific | GBR Mid-shelf | 1995 | 2013 | 15 | Absolute | 0.0121 | 0.0050 | **F =5.810, df = 1, 13, P = 0.032** |
|  |  |  |  |  |  |  | Relative | 0.0006 | 0.0002 | **F = 10.770, df = 1, 13, P = 0.006** |
| Acroporidae | *Isopora* | Indo-Pacific | GBR Mid-shelf | 1995 | 2013 | 15 | Absolute | -0.0105 | 0.0055 | F = 3.700, df = 1,13, P = 0.077 |
|  |  |  |  |  |  |  | Relative | -0.0002 | 0.0002 | F = 1.980, df = 1, 13, P = 0.183 |
| Incertae sedis | *Leptastrea* | Indo-Pacific | GBR Mid-shelf | 1995 | 2013 | 15 | Absolute | 0.0044 | 0.0006 | **F = 53.110, df = 1, 13, P < 0.001** |
|  |  |  |  |  |  |  | Relative | 0.0002 | 0.0000 | **F = 51.530, df = 1, 13, P < 0.001** |
| Merulinidae | *Phymastrea* | Indo-Pacific | GBR Mid-shelf | 1995 | 2013 | 15 | Absolute | 0.0090 | 0.0026 | **F = 11.650, df = 1, 13, P = 0.005** |
|  |  |  |  |  |  |  | Relative | 0.0004 | 0.0001 | **F = 14.120, df = 1, 13, P = 0.002** |
| Acroporidae | *Montipora* | Indo-Pacific | GBR Mid-shelf | 1995 | 2013 | 15 | Absolute | 0.0114 | 0.0129 | F = 0.780, df = 1,13, P = 0.394 |
|  |  |  |  |  |  |  | Relative | 0.0011 | 0.0005 | F = 4.310, df =1, 13, P = 0.058 |
| Agaricidae | *Pavona* | Indo-Pacific | GBR Mid-shelf | 1995 | 2013 | 15 | Absolute | 0.0125 | 0.0025 | **F = 25.540, df = 1, 13, P < 0.001** |
|  |  |  |  |  |  |  | Relative | 0.0005 | 0.0001 | **F = 34.410, df = 1, 13, P < 0.001** |
| Pocilloporidae | *Pocillopora* | Indo-Pacific | GBR Mid-shelf | 1995 | 2013 | 15 | Absolute | 0.0204 | 0.0085 | F = 5.740, df = 1,13, P = 0.032 |
|  |  |  |  |  |  |  | Relative | 0.0011 | 0.0002 | **F = 32.840, df = 1, 13, P < 0.001** |
| Poritidae | *Porites* | Indo-Pacific | GBR Mid-shelf | 1995 | 2013 | 15 | Absolute | 0.046 | 0.016 | **F = 8.8.084, df = 1, 13, P = 0.014** |
|  |  |  |  |  |  |  | Relative | 0.003 | 0.001 | **F = 7.106, df = 1, 13, P = 0.019** |
| Pocilloporidae | *Seriatopora* | Indo-Pacific | GBR Mid-shelf | 1995 | 2013 | 15 | Absolute | -0.0144 | 0.0102 | F = 2.010, df = 1,3, P = 0.189 |
|  |  |  |  |  |  |  | Relative | -0.0003 | 0.0003 | F = 1.300, df = 1, 13, P = 0.275 |
| Pocilloporidae | *Stylophora* | Indo-Pacific | GBR Mid-shelf | 1995 | 2013 | 15 | Absolute | 0.0021 | 0.0063 | F = 0.110, df = 1,13, P = 0.743 |
|  |  |  |  |  |  |  | Relative | 0.0003 | 0.0002 | F = 2.980, df = 1, 13, P = 0.108 |
| Acroporidae | *Acropora* | Indo-Pacific | GBR Outer shelf | 1995 | 2013 | 15 | Absolute | -0.5022 | 0.3876 | F = 1.680, df = 1, 13, P = 0.218 |
|  |  |  |  |  |  |  | Relative | -0.0071 | 0.0070 | F = 1.020, df = 1, 13, P = 0.331 |
| Merulinidae | *Echinopora* | Indo-Pacific | GBR Outer shelf | 1995 | 2013 | 15 | Absolute | -0.0031 | 0.0027 | F = 1.300, df = 1, 13, P = 0.275 |
|  |  |  |  |  |  |  | Relative | 0.0003 | 0.0001 | F = 6.400, df = 1, 13, P = 0.025 |
| Merulinidae | *Goniastrea* | Indo-Pacific | GBR Outer shelf | 1995 | 2013 | 15 | Absolute | -0.0034 | 0.0054 | F = 0.410, df = 1, 13, P = 0.534 |
|  |  |  |  |  |  |  | Relative | 0.0005 | 0.0003 | F = 3.590, df = 1, 13, P = 0.081 |
| Acroporidae | *Isopora* | Indo-Pacific | GBR Outer shelf | 1995 | 2013 | 15 | Absolute | -0.0122 | 0.0067 | F = 3.330, df = 1, 13, P = 0.091 |
|  |  |  |  |  |  |  | Relative | 0.0022 | 0.0010 | F = 4.610, df = 1, 13, P = 0.051 |
| Incertae sedis | *Leptastrea* | Indo-Pacific | GBR Outer shelf | 1995 | 2013 | 15 | Absolute | 0.0053 | 0.0009 | **F = 32.870, df = 1, 13, P < 0.001** |
|  |  |  |  |  |  |  | Relative | 0.0003 | 0.0001 | **F = 23.070, df = 1, 13, P < 0.001** |
| Merulinidae | *Phymastrea* | Indo-Pacific | GBR Outer shelf | 1995 | 2013 | 15 | Absolute | 0.0034 | 0.0030 | F =1.230, df = 1, 13, P = 0.287 |
|  |  |  |  |  |  |  | Relative | 0.0004 | 0.0002 | **F = 5.600, df = 1, 13, P = 0.034** |
| Acroporidae | *Montipora* | Indo-Pacific | GBR Outer shelf | 1995 | 2013 | 15 | Absolute | 0.0200 | 0.0096 | F = 4.290, df = 1, 13, P = 0.059 |
|  |  |  |  |  |  |  | Relative | 0.0027 | 0.0006 | **F = 19.660, df =1, 13, P = 0.001** |
| Agaricidae | *Pavona* | Indo-Pacific | GBR Outer shelf | 1995 | 2013 | 15 | Absolute | 0.0079 | 0.0020 | **F = 16.010, df = 1, 13, P = 0.002** |
|  |  |  |  |  |  |  | Relative | 0.0005 | 0.0001 | **F = 18.840, df = 1, 13, P = 0.001** |
| Pocilloporidae | *Pocillopora* | Indo-Pacific | GBR Outer shelf | 1995 | 2013 | 15 | Absolute | -0.0639 | 0.0149 | **F = 18.290, df = 1, 13, P = 0.001** |
|  |  |  |  |  |  |  | Relative | -0.0002 | 0.0005 | F = 0.090, df = 1, 13, P = 0.766 |
| Poritidae | *Porites* | Indo-Pacific | GBR Outer shelf | 1995 | 2013 | 15 | Absolute | -0.001 | 0.011 | F = 0.003, df = 1, 13, P = 0.961 |
|  |  |  |  |  |  |  | Relative | 0.003 | 0.001 | **F = 8.806, df = 1, 13, P = 0.011** |
| Pocilloporidae | *Seriatopora* | Indo-Pacific | GBR Outer shelf | 1995 | 2013 | 15 | Absolute | 0.0091 | 0.0032 | **F = 8.420, df = 1, 13, P = 0.012** |
|  |  |  |  |  |  |  | Relative | 0.0010 | 0.0003 | **F = 14.230, df = 1, 13, P = 0.002** |
| Pocilloporidae | *Stylophora* | Indo-Pacific | GBR Outer shelf | 1995 | 2013 | 15 | Absolute | -0.0320 | 0.0045 | **F = 50.870, df = 1, 13, P < 0.001** |
|  |  |  |  |  |  |  | Relative | 0.0000 | 0.0005 | F < 0.001, df = 1, 13, P = 0.991 |

Table S3. Fossil Data. Slope, standard error of slope, *P* value, and sample size (N, number of times sampled during the time period specified) for each taxon (family and genus) from the fossil record (0.125 to 6.8 Ma). Samples were extracted from outcrop exposures at 70 localities through four Plio-Pleistocene sequences: Costa Rica [31]; Curacao [32]; Dominican Republic [33]; and, Jamaica [34]. Rank abundance shown from most–least abundant, Extinction shows whether the genus became extinct.

| **Family** | **Genus** | **N** | **Slope** | **SE of slope** | ***P* value** | **Rank Abundance (most - least)** | **Extinct?** |
| --- | --- | --- | --- | --- | --- | --- | --- |
| Acroporidae | *Acropora* | 70 | 2.650 | 0.816 | 0.000 | 3.00 | N |
| Agariciidae | *Agaricia* | 70 | 1.769 | 0.614 | 0.010 | 7.00 | Y |
| Mussidae | *Antillia* | 70 | -0.373 | 0.197 | 0.060 | 30.46 | Y |
| Trachyphylliidae | *Antillophyllia* | 70 | -0.174 | 0.094 | 0.070 | 35.37 | Y |
| Merulinidae | *Caulastraea* | 70 | 0.601 | 0.274 | 0.030 | 17.67 | Y |
| Mussidae | *Colpophyllia* | 70 | 0.756 | 0.139 | 0.000 | 20.62 | N |
| Meandrinidae | *Dichocoenia* | 70 | 0.359 | 0.164 | 0.030 | 18.66 | N |
| Mussidae | *Diploria* | 70 | 1.212 | 0.381 | 0.000 | 9.81 | N |
| Meandrinidae | *Eusmilia* | 70 | 0.225 | 0.104 | 0.030 | 32.42 | N |
| Mussidae | *Favia* | 70 | -0.046 | 0.163 | 0.780 | 31.44 | N |
| Agariciidae | *Gardineroseris* | 70 | -0.141 | 0.050 | 0.010 | 38.32 | Y |
| Poritidae | *Goniopora* | 70 | -0.646 | 0.183 | 0.000 | 27.51 | Y |
| Faviidae | *Hadrophyllia* | 70 | 0.006 | 0.067 | 0.930 | 34.39 | Y |
| Agariciidae | *Helioseris* | 70 | 0.278 | 0.387 | 0.480 | 21.61 | N |
| Mussidae | *Isophyllia* | 70 | -0.052 | 0.046 | 0.260 | 37.34 | N |
| Acroporidae | *Isopora* | 70 | -0.080 | 0.411 | 0.850 | 16.69 | Y |
| Agariciidae | *Leptoseris* | 70 | -0.924 | 0.182 | 0.000 | 24.56 | N |
| Astrocoeniidae | *Madracis* | 70 | 0.694 | 0.371 | 0.070 | 11.77 | N |
| Mussidae | *Manicina* | 70 | -1.644 | 0.631 | 0.010 | 5.00 | N |
| Meandrinidae | *Meandrina* | 70 | -0.430 | 0.248 | 0.090 | 19.64 | N |
| Montastraeidae | *Montastraea* | 70 | 1.156 | 0.305 | 0.000 | 2.00 | N |
| Mussidae | *Mussa* | 70 | 0.028 | 0.035 | 0.420 | 36.36 | N |
| Mussidae | *Mussismilia* | 70 | 0.028 | 0.156 | 0.860 | 29.47 | Y |
| Mussidae | *Mycetophyllia* | 70 | 0.505 | 0.126 | 0.000 | 26.52 | N |
| Mussidae | *Obicella* complex | 70 | -0.473 | 0.807 | 0.560 | 10.79 | N |
| Agariciidae | *Pavona* | 70 | -0.585 | 0.288 | 0.050 | 23.57 | Y |
| Meandrinidae | *Placocyathus* | 70 | -1.518 | 0.695 | 0.030 | 8.00 | N |
| Pocilloporidae | *Pocillopora* | 70 | -0.383 | 0.291 | 0.190 | 25.54 | Y |
| Poritidae | *Porites* branching | 70 | 0.394 | 0.413 | 0.340 | 5.00 | N |
| Poritidae | *Porites* massive | 70 | -1.136 | 0.359 | 0.000 | 9.00 | N |
| Psammocoridae | *Psammacora* | 70 | -0.857 | 0.263 | 0.000 | 28.49 | Y |
| Mussidae | *Scolmyia* | 70 | 0.029 | 0.074 | 0.700 | 33.41 | N |
| Siderastreidae | *Siderastrea* | 70 | 0.442 | 0.300 | 0.140 | 14.72 | N |
| Incertae sedis | *Solenastrea* | 70 | -0.887 | 0.289 | 0.000 | 15.71 | N |
| Astrocoeniidae | *Stephanocoenia* | 70 | 0.896 | 0.353 | 0.010 | 13.74 | N |
| Pocilloporidae | *Stylophora* | 70 | -1.706 | 0.880 | 0.060 | 1.00 | Y |
| Faviidae | *Thysanus* | 70 | 0.113 | 0.223 | 0.610 | 22.59 | Y |
| Trachyphylliidae | *Trachyphyllia* | 70 | -1.758 | 0.580 | 0.000 | 12.76 | Y |
| Agariciidae | *Undaria* | 70 | 1.653 | 0.571 | 0.010 | 4.00 | N |

Table S4. Summary of changes in coral cover (% y^-1^) on absolute and relative scales as displayed in Figs. 1 and S2. Entries are organized by quadrant on the graphic of absolute versus relative change, region (Indo-Pacific and Caribbean), family (after 24), and genus. Mean and SD (where N > 1) were based on location replicates (N, Table S1). Abundance Rank gives the relative abundance based on percentage cover averaged among all times and sites within regions. Cover ranged from 0.002% (*Mussa*, rank 1) to 15.538% (*Orbicella*, rank 16) in the Caribbean, and from 0.002% (*Stylocoeniella*, rank 1) to 7.031% (*Acropora*, rank 41) in the Indo-Pacific. For raw data see Table S2 and <http://knb.ecoinformatics.org/knb/metacat/nceas.973/knb>.

|  |  |  |  | Absolute (% y^-1^) |  | Relative (% y^-1^) |  |  |  |
| --- | --- | --- | --- | --- | --- | --- | --- | --- | --- |
| **Quadrant** | **Region** | **Family** | **Genus** | **Mean** | **SD** | **Mean** | **SD** | **N** | **Rank** |
| F-corals | Caribbean | Merulinidae | *Orbicella* | -0.5730 | 0.5024 | -0.1494 | 0.3193 | 9 | 16 |
| F-corals | Caribbean | Montastraeidae | *Montastraea* | -0.0550 | 0.0717 | -0.0118 | 0.0599 | 9 | 12 |
| F-corals | Caribbean | Mussidae | *Colpophyllia* | -0.0269 | 0.0352 | -0.0769 | 0.0946 | 2 | 10 |
| F-corals | Caribbean | Mussidae | *Diploria* | -0.0249 | 0.0208 | -0.0021 | 0.0239 | 8 | 11 |
| F-corals | Caribbean | Acroporidae | *Acropora* | -0.0100 |  | -0.0400 |  | 1 | 8 |
| F-corals | Caribbean | Agaricidae | *Agaricia* | -0.0094 | 0.0678 | -0.0189 | 0.2024 | 8 | 13 |
| F-corals | Caribbean | Mussidae | *Favia* | -0.0060 |  | -0.0340 |  | 1 | 5 |
| F-corals | Caribbean | Mussidae | *Manicina* | -0.0030 |  | -0.0090 |  | 1 | 3 |
| F-corals | Caribbean | Mussidae | *Scolymia* | -0.0010 |  | -0.0040 |  | 1 | 2 |
| W-corals | Caribbean | Poritidae | *Porites* | -0.0106 | 0.0508 | 0.1716 | 0.2361 | 9 | 15 |
| W-corals | Caribbean | Meandrinidae | *Dichocoenia* | -0.0001 | 0.0032 | 0.0110 | 0.0272 | 6 | 4 |
| S-corals | Caribbean | Mussidae | *Mussa* | 0.0020 |  | 0.0120 |  | 1 | 1 |
| S-corals | Caribbean | Meandrinidae | *Eusmilia* | 0.0030 |  | 0.0140 |  | 1 | 7 |
| S-corals | Caribbean | Meandrinidae | *Meandrina* | 0.0063 | 0.0126 | 0.0048 | 0.0081 | 6 | 9 |
| M-corals | Caribbean | Siderastreidae | *Siderastrea* | 0.0091 | 0.0576 | -0.0477 | 0.2155 | 9 | 14 |
| M-corals | Caribbean | Astrocoeniidae | *Stephanocoenia* | 0.0035 | 0.0053 | -0.0127 | 0.0321 | 6 | 6 |
|  |  |  |  |  |  |  |  |  |  |
| F-corals | Indo-Pacific | Pocilloporidae | *Pocillopora* | -0.4385 | 1.0569 | -0.4172 | 0.6900 | 11 | 39 |
| F-corals | Indo-Pacific | Acroporidae | *Acropora* | -0.3656 | 0.6052 | -0.8688 | 1.5719 | 10 | 41 |
| F-corals | Indo-Pacific | Acroporidae | *Montipora* | -0.1948 | 0.3593 | -0.6952 | 1.5837 | 11 | 37 |
| F-corals | Indo-Pacific | Poritidae | *Porites* | -0.1040 | 0.5128 | 0.7012 | 1.4832 | 11 | 40 |
| F-corals | Indo-Pacific | Merulinidae | *Leptoria* | -0.0890 | 0.1343 | -0.2816 | 0.4307 | 2 | 15 |
| F-corals | Indo-Pacific | Merulinidae | *Scapophyllia* | -0.0758 |  | -0.2551 |  | 1 | 16 |
| F-corals | Indo-Pacific | Fungidae | *Fungia* | -0.0573 | 0.1171 | -0.1671 | 0.3066 | 5 | 25 |
| F-corals | Indo-Pacific | Incertae sedis | *Leptastrea* | -0.0390 | 0.0794 | -0.0436 | 0.1838 | 9 | 30 |
| F-corals | Indo-Pacific | Euphylliidae | *Galaxea* | -0.0312 | 0.0554 | -0.4612 | 0.6677 | 2 | 35 |
| F-corals | Indo-Pacific | Acroporidae | *Astreopora* | -0.0300 | 0.0217 | -0.0308 | 0.0321 | 3 | 21 |
| F-corals | Indo-Pacific | Lobophyllidae | *Acanthastrea* | -0.0295 | 0.0120 | -0.0025 | 0.0120 | 2 | 10 |
| F-corals | Indo-Pacific | Pocilloporidae | *Stylophora* | -0.0179 | 0.0179 | -0.0687 | 0.1375 | 4 | 33 |
| F-corals | Indo-Pacific | Merulinidae | *Goniastrea* | -0.0086 | 0.8147 | -0.5741 | 1.8608 | 6 | 29 |
| F-corals | Indo-Pacific | Merulinidae | *Hydnopora* | -0.0063 | 0.1384 | -0.2388 | 0.2160 | 3 | 24 |
| F-corals | Indo-Pacific | Pocilloporidae | *Plesiastrea* | -0.0030 | 0.0037 | -0.0109 | 0.0139 | 2 | 9 |
| F-corals | Indo-Pacific | Lobophyllidae | *Echinophyllia* | -0.0014 | 0.0019 | -0.1438 | 0.2036 | 2 | 2 |
| F-corals | Indo-Pacific | Dendrophyllidae | *Tubastrea* | -0.0010 |  | -0.0030 |  | 1 | 5 |
| M-corals | Indo-Pacific | Agaricidae | *Pachyseris* | 0.0002 |  | -0.0034 |  | 1 | 12 |
| M-corals | Indo-Pacific | Merulinidae | *Echinopora* | 0.0009 | 0.0443 | -0.0063 | 0.1095 | 5 | 34 |
| M-corals | Indo-Pacific | Merulinidae | *Favites* | 0.6344 | 0.6731 | -0.0162 | 2.5163 | 3 | 38 |
| S-corals | Indo-Pacific | Agaricidae | *Gardineroseris* | 0.0003 |  | 0.0010 |  | 1 | 4 |
| S-corals | Indo-Pacific | Lobophylliidae | *Oxypora* | 0.0020 | 0.0026 | 0.0032 | 0.0040 | 2 | 3 |
| S-corals | Indo-Pacific | Dendrophyllidae | *Turbinaria* | 0.0023 | 0.0018 | 0.0033 | 0.0039 | 2 | 11 |
| S-corals | Indo-Pacific | Poritidae | *Alveopora* | 0.0030 |  | 0.0110 |  |  | 13 |
| S-corals | Indo-Pacific | Merulinidae | *Oulophyllia* | 0.0039 |  | 0.0099 |  | 1 | 6 |
| S-corals | Indo-Pacific | Pocilloporidae | *Seriatopora* | 0.0041 | 0.0231 | 0.0289 | 0.0421 | 5 | 26 |
| S-corals | Indo-Pacific | Agaricidae | *Pavona* | 0.0046 | 0.0986 | 0.1242 | 0.1567 | 9 | 32 |
| S-corals | Indo-Pacific | Coscinaraeidae | *Coscinaraea* | 0.0068 | 0.0082 | 0.0172 | 0.0201 | 2 | 7 |
| S-corals | Indo-Pacific | Faviidae | *Herpolitha* | 0.0120 | 0.0182 | 0.0254 | 0.0370 | 3 | 18 |
| S-corals | Indo-Pacific | Pocilloporidae | *Stylocoeniella* | 0.0139 |  | 0.0356 |  | 1 | 1 |
| S-corals | Indo-Pacific | Merulinidae | *Cyphastrea* | 0.0503 | 0.0634 | 0.0034 | 0.0228 | 3 | 14 |
| S-corals | Indo-Pacific | Poritidae | *Synarea* | 0.0540 |  | 0.1870 |  | 1 | 27 |
| S-corals | Indo-Pacific | Lobophylliidae | *Symphyllia* | 0.0734 |  | 0.1645 |  | 1 | 22 |
| S-corals | Indo-Pacific | Lobophylliidae | *Lobophyllia* | 0.0890 | 0.1245 | 0.2057 | 0.2901 | 2 | 8 |
| S-corals | Indo-Pacific | Merulinida | *Merulina* | 0.1450 |  | 0.3659 |  | 1 | 17 |
| S-corals | Indo-Pacific | Poritidae | *Goniopora* | 0.3000 | 0.5932 | 1.2569 | 2.4299 | 3 | 19 |
| S-corals | Indo-Pacific | Merulinida | *Platygyra* | 0.4398 | 0.7021 | 1.0752 | 1.6424 | 3 | 31 |
| S-corals | Indo-Pacific | Merulinidae | *Dipsastraea* | 0.6383 | 0.9941 | 1.2743 | 2.6952 | 3 | 28 |
| W-corals | Indo-Pacific | Merulinidae | *Phymastrea* | -0.0237 | 0.0465 | 0.2260 | 0.5700 | 8 | 20 |
| W-corals | Indo-Pacific | Acroporidae | *Isopora* | -0.0101 | 0.0023 | 0.0006 | 0.0014 | 3 | 36 |
| W-corals | Indo-Pacific | Psammocoridae | *Psammocora* | -0.0094 | 0.0344 | 0.0851 | 0.2152 | 4 | 23 |

Table S5. Model parameters; only those related to coral characteristics have ranges (minimum and maximum values). Parameter values informed by [44] and the expert opinion of the present authors (note that the range for  includes all possible values); tests of different parameter ranges did not alter the results of global sensitivity analyses (GSA). DHM = degree heating months [48].

| Parameter name | Symbol | Minimum | Maximum | Units |
| --- | --- | --- | --- | --- |
|  |  | value | value |  |
| Coral recruitment | *r_A_* | 0.0005 | 0.05 | time^-1^ |
| Coral maturation | *a* | 0.2 | 0.65 | time^-1^ |
| Coral growth | *g* | 0.01 | 0.4 | time^-1^ |
| Juvenile coral mortality | *d_R_* | 0.25 | 1.5 | time^-1^ |
| Adult coral mortality/shrinkage | *d_A_* | 0.002 | 0.4 | time^-1^ |
| Dispersal |  | 0 | 1 | unitless (proportion larvae dispersing) |
| Coral overgrowth resistance to macroalgae |  | 0 | 0.4 | time^-1^ |
| Herbivore habitat provision |  | 0 | 4 | (proportional area adult cover) ^-1^ |
| Bleaching resistance |  | 0 | 0.25 | proportional mortalityDHM^-1^ |
| Disturbance intercept | *b* | 0.375 | | proportional mortality |
| Macroalgae growth and recruitment | *r_M_* | 0.4 | | time^-1^ |
| Baseline herbivory | *h_b_* | 0.2 | | time^-1^ |
| Supplemented herbivory | *h_s_* | 0.4 | | time^-1^ |
| Number of patches | *n* | 10 | | number |
| Disturbance standard deviation |  | 1 | | DHM |

Figure S1. Scatterplots displaying the relationships between changes in relative abundance and dominance in the community for coral genera [Table S4]) in the US Virgin Islands, and Belize (A) and Moorea, the GBR, Kenya, Hawaii, and Taiwan (B). Rank abundances were determined as in Table S3, and are displayed from rare (left) to abundant (right); families color-coded as shown.


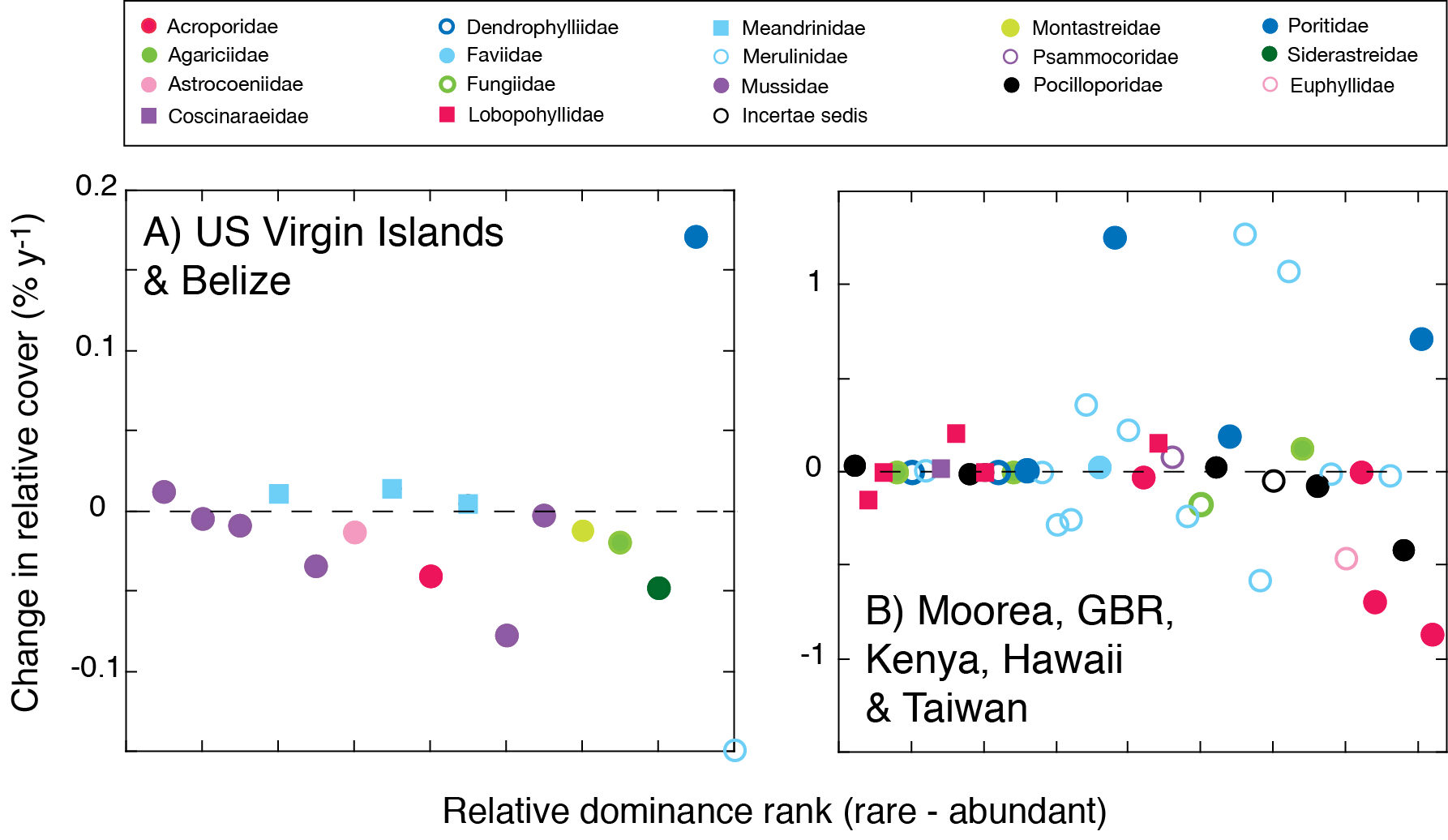


Figure S2. Changes in past coral communities as represented by the fossil record between 6.8 and 0.125 Ma displaying changes in relative abundance (% Myr^-1^) with genus-level resolution (Table S3). Scatterplot displays changes in relative abundance by genus against relative dominance (ranked from low [left] to high [right]) in the community, with family color-coded as shown; open symbols are taxa that became extinct, closed symbols are those that survived, both for the Caribbean.


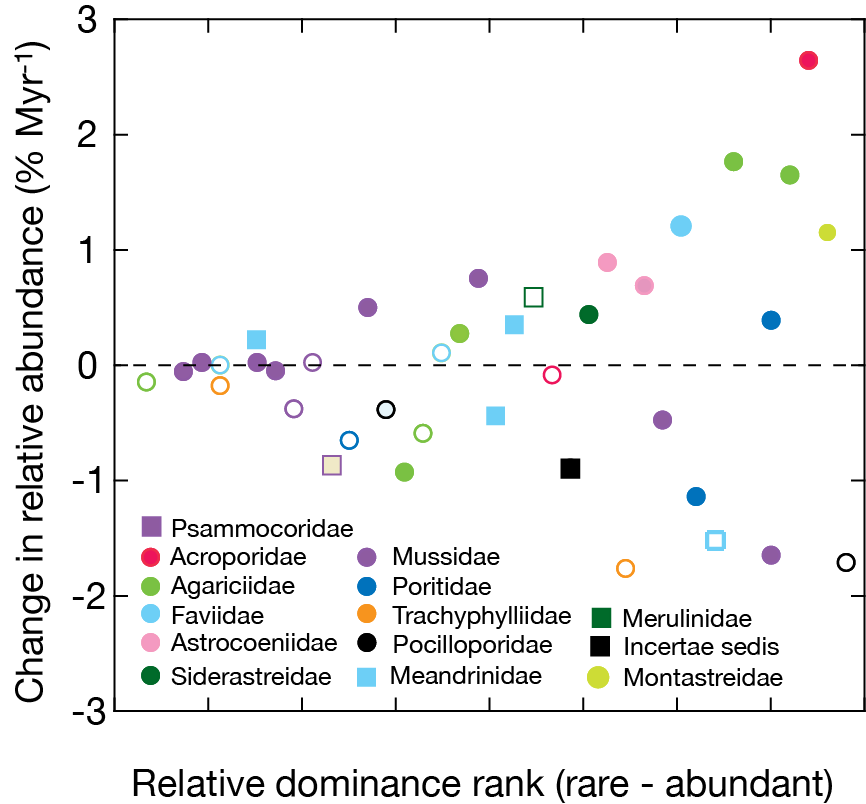


Figure S3. Boxplots showing proportion of coral cover at the end of model simulations using AR5 climate model output. The same y-axis scale is used for comparison with Fig. 6 in the main text. Simulated corals have a range of possible outcomes using the AR4 output, but final cover does not exceed 10% in model runs under any AR5 climate scenario and mean coral cover is zero on average.


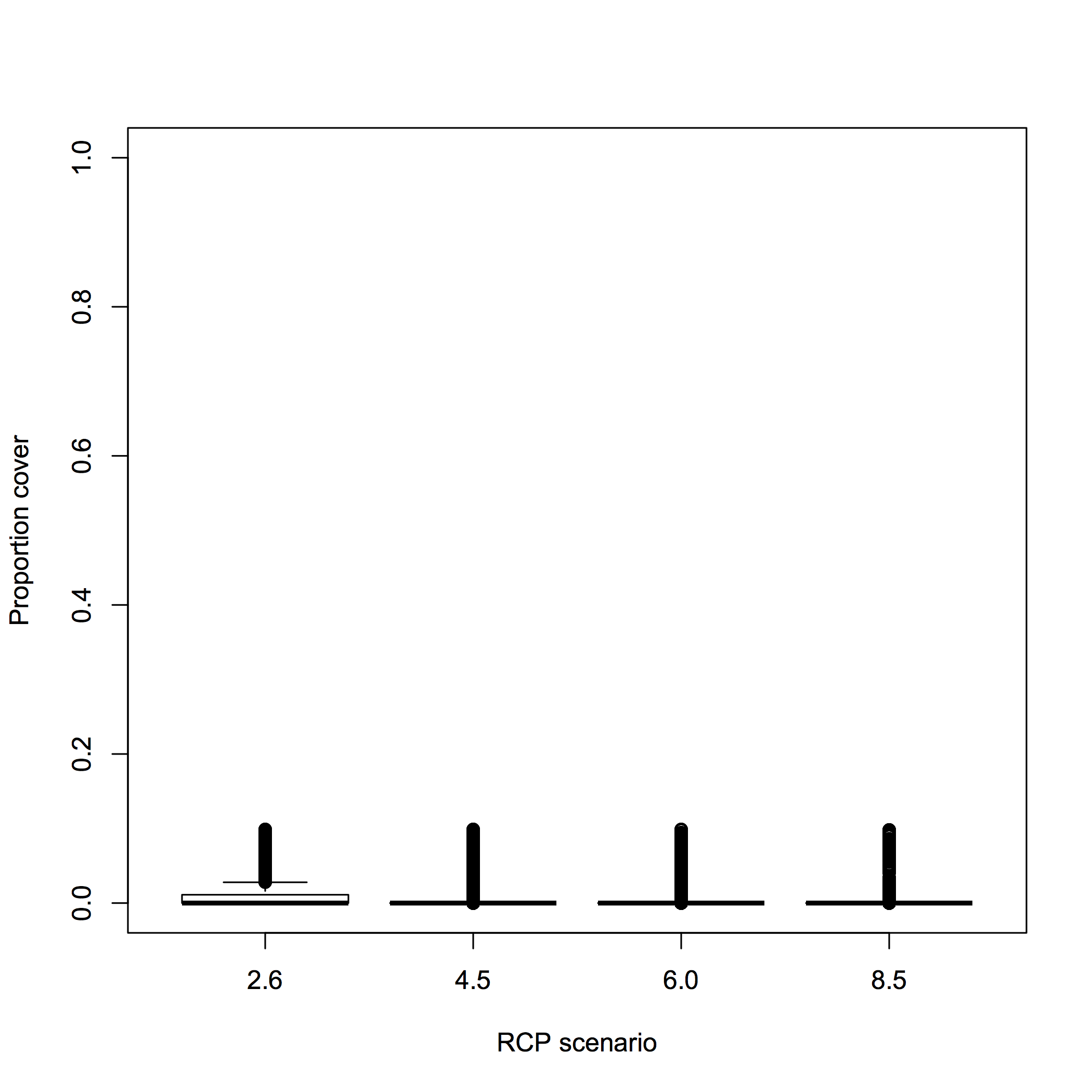


Figure S4. Normalized importance values for ecological processes for future climate change scenarios using output from the fifth IPCC assessment report. The RCP scenarios describe the amount of radiative forcing in the year 2100 relative to pre-industrial values (W/m^2^). These GSA importance values represent the results of 1,000 simulations for each RCP scenario, each with a unique set of randomly drawn parameter values, analyzed for which parameters were most influential for coral persistence. Large increases in mean and maximum sea surface temperatures in the AR5 report and the lack of a "commit" scenario lead to greater importance values for bleaching resistance, but adult coral mortality and coral growth are still more important for locations or time periods with milder bleaching events.


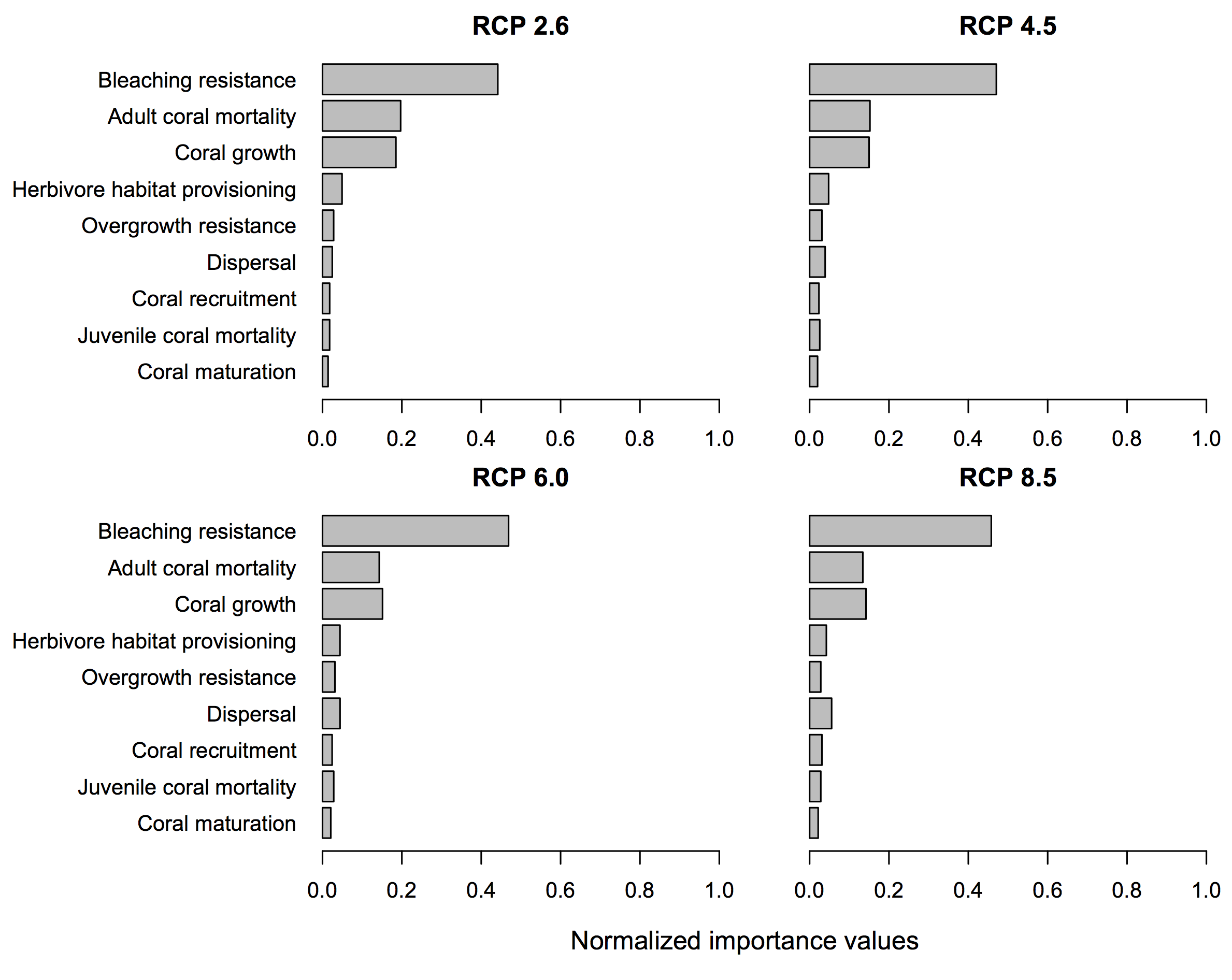


**References for Supporting Information**

78. Edmunds PJ (2013) Decadal-scale changes in the community structure of coral reefs of St. John, US Virgin Islands. Mar Ecol Prog Ser 489: 107-123

79. Smith TB, Nemeth RS, Blondeau J, Calnan JM, Kadison E, et al. (2008) Assessing coral reef health across onshore to offshore stress gradients in the US Virgin Islands. Mar Poll Bull 56: 1983-1991.

80. McClanahan TR, Muthiga NA, Coleman RA (2011) Testing for top-down control: can post-disturbance fisheries closures reverse algal dominance? Aquat Conser 21: 658-675.

81. http://mcr.lternet.edu/

82. Coles SL, Brown EK (2007) Twenty-five years of change in coral coverage on a hurricane impacted reef in Hawai‘i: the importance of recruitment. Coral Reefs 26:705-717.

83. McClanahan TR (2008) Response of the coral reef benthos and herbivory to fishery closure management and the 1998 ENSO disturbance. Oecologia 155: 169-177. doi:10.1007/s00442-007-0890-0.

84. Osborne K, Dolman A, Burgess S, Johns K (2011) Disturbance and the dynamics of coral cover on the Great Barrier Reef (1995-2009). PLoS ONE 6: e17516.

85. AIMS: http://www.aims.gov.au/documents/30301/e055df14-24d0-40ca-b46d-4ae1988da07c
